# Artificial intelligence, green innovation efficiency, and public health benefits: evidence from China’s pilot zones with a DID approach

**DOI:** 10.3389/fpubh.2025.1713295

**Published:** 2025-11-18

**Authors:** Zhihong Zhang, Ming Zhang, Xing Xu

**Affiliations:** 1 School of Economics and Management, Weifang University of Science and Technology, Weifang, Shandong, China; 2 Department of Economics and Management, Nanjing Agricultural University, Nanjing, China; 3 Department of Economics and Management, Shanghai Institute of Technology, Shanghai, China

**Keywords:** artificial intelligence, green innovation, green R&D efficiency, GreenInnovation output efficiency, public health benefits

## Abstract

**Introduction:**

China has established Artificial Intelligence Innovation and Development Pilot Zones (AIDP) to promote industrial upgrading and digital transformation. This study evaluates whether these AI oriented policies enhance firm-level green innovation efficiency (GIE) and generate potential environmental and health benefits.

**Methods:**

Using panel data of Chinese firms from 2011 to 2022, we employed a difference-in-differences (DID) design exploiting the staggered implementation of AIDP as a quasi-experiment. The estimation identifies the causal effects of AIDP on green R&D efficiency and green innovation output efficiency, measured by DEA-based indicators and patent productivity.

**Results:**

The DID estimates show that AIDP increases firms’ green R&D efficiency by approximately 1.7% and green innovation output efficiency by 1.4% relative to non-pilot firms, corresponding to the DID coefficients of 0.017 and 0.014. These effects are primarily driven by enhanced AI hardware investment, improved asset liquidity, and strengthened ESG performance, which together boost firms’ digital capability, operational efficiency, and sustainability orientation. Mechanism analysis further indicates that these improvements reduce pollution intensity and support cleaner production processes, suggesting potential environmental and health benefits inferred from pollution reduction. Heterogeneity analysis reveals that the treatment effects are larger among non-state-owned enterprises, manufacturing firms, and labor-intensive sectors, underscoring the importance of tailoring interventions to sectoral and institutional contexts.

**Conclusion:**

AIDP generates economically and statistically significant improvements in green innovation efficiency, confirming the potential of AI oriented policies to promote sustainable industrial transformation. By fostering green technologies that cut emissions and reduce industrial pollution, these policies deliver dual dividends: advancing environmental sustainability and supporting public health. The findings highlight the importance of expanding pilot zones, integrating digital initiatives with green finance, and investing in digital infrastructure to maximize policy effectiveness and generate broader environmental and health benefits.

## 1 Introduction

The rapid development of artificial intelligence (AI) is transforming industries and the digital economy. This raises an important question: can AI policies improve firms’ green innovation efficiency (GIE) while also bringing public health benefits? Although many countries have introduced AI related strategies, such as the EU’s “AI Act,” the Green Deal Industrial Plan, and Japan’s Society 5.0, they are still exploring how to align AI development with sustainability and health goals. Against this global backdrop, China’s Artificial Intelligence Innovation and Development Pilot Zones (AIDP) represent one of the earliest and most comprehensive national experiments integrating AI policy with green and inclusive growth goals. Studying AIDP offers a timely opportunity to examine whether AI based industrial policies can promote environmental and health improvements beyond traditional technological upgrading. As countries confront the dual challenge of digital transformation and carbon reduction, evidence from AIDP provides lessons for designing AI governance frameworks that promote innovation, sustainability, and social welfare together. Overall, this context underscores the global importance of examining China’s AIDP as both a domestic reform initiative and a reference for international AI governance. The investigation of AIDP is both nationally significant and globally relevant, especially for achieving carbon neutrality, advancing the Healthy China 2030 agenda, and contributing to global discussions on AI supported sustainable transitions. By linking the digital and green transition with public health outcomes, this study situates AIDP within a broader international context that includes policy initiatives such as the EU’s AI Act, the Green Deal Industrial Plan, and Japan’s Society 5.0, as well as foundational research by Kusche (1), and Holroyd (2). Since 2019, China has established Artificial Intelligence Innovation and Development Pilot Zones (AIDP) to promote AI applications across sectors. Existing studies have examined AI’s impact on innovation and economic performance, but the causal effects of government led AI initiatives like AIDP on firm’s level GIE and related environmental and health outcomes remain unclear. Filling this gap is crucial for designing policies that link technological progress with sustainability and health goals, helping reduce pollution related diseases through cleaner production (3, 4). AIDP serves as a unique quasi-experiment that helps identify how AI policies affect green innovation and pollution intensity, moving beyond the descriptive evidence of earlier studies. Furthermore, while most existing literature focuses on technological or economic effects, few examine policy-induced causal impacts or consider public health benefits. This study fills that gap by integrating digital, environmental, and health dimensions within a unified analytical framework. To this end, we apply a difference-in-differences (DID) approach to estimate the effects of AIDP, providing empirical insights relevant to both academic understanding and policy design in other countries pursuing AI based sustainability strategies.

Before turning to the empirical model, the following literature review synthesizes existing findings and clarifies the theoretical linkages between AI, GIE, and health outcomes. Our literature review aims to establish a coherent theoretical foundation for examining how artificial intelligence (AI) influences green innovation efficiency (GIE) and its broader public health implications. We organize the existing research around two main theoretical perspectives: technological innovation and digital transformation theory, and the environmental regulation and benefit framework. The first theoretical perspective explains how AI enhances firms’ innovation efficiency through automation, knowledge diffusion, and resource reallocation (5–7), while the second framework links green innovation to environmental and health outcomes (8–11). Building on these perspectives, we categorize prior studies into three strands: research on GIE and its determinants, studies on AI and sustainable transformation, and work connecting environmental improvement with public health benefits. This structure avoids topic-jumping and clarifies the causal and conceptual pathways among AI, green innovation, and public health.

The first strand of literature focuses on green innovation efficiency, which reflects not only firms’ technological capability but also their ability to transform innovation inputs into environmentally and socially beneficial outcomes. GIE is shaped by factors such as financial incentives, environmental regulation, and regional collaboration (12–14). Studies show that policies like green finance reforms and low-carbon city initiatives can significantly enhance green innovation (15–18). Recent evidence by Jiang and Yuan (19) further demonstrates that while green credit expansion in China positively affects the scale of real economic activity, it may undermine efficiency by distorting innovation incentives, highlighting the tension between financial support and sustainable technological upgrading. This finding suggests that financial policies should balance growth and innovation efficiency to ensure that green credit promotes real green transformation instead of reducing innovation quality. However, much of this research remains fragmented, often addressing technological efficiency or environmental outcomes separately rather than within an integrated framework. Beyond simple patent counts, methods such as DEA, the Super-SBM model, and the Malmquist index enable a joint assessment of desirable and undesirable outputs (16, 17). For example, the Super-SBM model captures pollution and inefficiency, providing a realistic estimation of trade-offs between economic inputs and environmental protection (20). These approaches are essential because they link technological progress to tangible environmental and health improvements, including emission reductions, lower pollutant exposure, and enhanced population well-being (21). Complementary spatial network studies, such as Hu et al. (22), reveal that low carbon patent collaboration in China forms complex multi-layered regional networks shaped by economic development, local scientific capacity, and low carbon awareness, suggesting that innovation efficiency is embedded within broader spatial and institutional contexts that ultimately influence environmental and health outcomes. However, most existing studies remain descriptive or focus on specific sectors, without testing whether AI driven industrial policies can shape these dynamics systematically. Furthermore, international studies highlight that green innovation is not only a technological outcome but also a social process with cross-border externalities (22, 23), underscoring the importance of a global perspective in understanding AI supported green transitions.

These insights motivate a two-stage analytical framework that links innovation inputs, processes, and outcomes. Building on these insights, we use a two stage analytical framework to evaluate GIE. The first stage measures how efficiently R&D inputs, such as personnel and spending, are converted into outputs like green patents. The second stage assesses how these outputs translate into final outcomes, including sales revenue, energy savings, and pollution reduction. This framework captures the full innovation value chain, from knowledge creation to commercialization, under environmental constraints. By integrating these two stages, it aligns with international research on eco-efficiency and innovation diffusion and underscores that improvements in GIE can directly reduce the health burden of pollution exposure. However, prior studies have not examined the dual linkage between AI policy, green innovation efficiency, and population health within a unified empirical setting, leaving an opportunity to bridge the digital economy and public health literatures.

Another strand of research investigates the relationship between AI and sustainable transformation. A growing body of evidence connects AI adoption to sustainability outcomes, emphasizing mechanisms such as optimized resource allocation, reduced production costs, and accelerated R&D productivity (24, 25). At the firm level, AI adoption enhances total factor productivity, strengthens knowledge coupling, and improves commercialization capabilities (24–27). For instance, Tian et al. (24) show that AI promotes manufacturing green innovation through knowledge integration, while Yin et al. (25) find stronger effects in regions with more advanced digital infrastructure and governance. Wang et al. (26) report that AI adoption in energy enterprises enhances innovation outcomes, particularly when supported by ESG practices, and Hussain et al. (27) demonstrate that financial analysts amplify AI’s positive influence by mitigating information asymmetry. Some studies further reveal nonlinear effects, such as a U shaped relationship between AI development and energy transition (28). Hu et al. (22) extend this discussion by examining innovation networks in the advanced medical equipment industry and show that regional innovation systems such as the Yangtze River Delta exhibit dense, polycentric AI driven collaborative networks that enhance technological diffusion and industrial upgrading. This evidence illustrates that digital collaboration and AI supported innovation ecosystems can strengthen regional public health resilience, thereby aligning digital transformation with health oriented industrial development. Similarly, Zhang et al. (19) highlight that the structural configuration of multinational R&D networks affects local knowledge creation, indicating that concentrated cross border collaborations can hinder host country innovation efficiency unless supported by dense domestic networks. These findings collectively suggest that the effectiveness of AI and digital innovation policies depends critically on network structures that enable broad based knowledge diffusion and inclusive capacity building.

Beyond the Chinese context, international studies reinforce the transformative potential of AI within global sustainability frameworks. Acemoglu and Restrepo (29) highlight how AI driven automation reshapes productivity and labor reallocation (30). Agrawal et al. (30) explore the role of AI in scientific discovery and innovation efficiency (31). Wang et al. (32) examine AI’s environmental impacts across OECD economies, and Bocean and Vărzaru (32) link technological transformation with sustainable development and public health (33). Taken together with the recent Chinese evidence (19, 34–36), the literature increasingly converges on a systems-oriented understanding of AI’s role in promoting green innovation efficiency: AI driven collaboration networks, green finance instruments, and digital infrastructure jointly shape the diffusion of green technologies and the realization of health benefits through pollution reduction and industrial resilience. These findings collectively suggest that the effectiveness of AI depends on complementary policy frameworks, such as the European Union’s AI Act, the Green Deal Industrial Plan, and Japan’s Society 5.0, which integrate digital innovation with environmental and social goals. Building on this international literature, our study positions China’s Artificial Intelligence Innovation and Development Pilot Zones (AIDP) as a representative policy experiment of AI supported green transition, providing evidence relevant beyond the Chinese context.

This study contributes to the literature by providing causal evidence on how government initiated AI initiatives, specifically the AIDP, affect firms’ green innovation efficiency (GIE) across both R&D and output dimensions. The core contribution lies in establishing the direct link between AI policy and firm-level GIE. While the study also conceptually discusses the potential public health benefits arising from improved green innovation, this channel is treated as a secondary, inferential implication rather than an empirically tested pathway. By situating China’s experience within the broader global policy context, this research enhances the international relevance of AI policy evaluation and offers policy insights for economies pursuing AI based sustainability transitions.

Conceptually, AIDP influences firm-level GIE through several reinforcing mechanisms, including increased AI hardware investment, optimized capital structure, improved liquidity, and stronger ESG performance. These mechanisms reinforce the synergy between digital and green innovation by enabling technology upgrading, efficiency enhancement, and sustainability governance, which jointly generate environmental and potential health benefits. By integrating these channels, our study overcomes the fragmentation in previous work and underscores the cumulative effect of AI based policy on economic upgrading, environmental quality, and human well-being. To identify causal impacts, we exploit the establishment of AIDP as a quasi-experiment and apply a difference in differences (DID) framework using firm level panel data from 2011 to 2022. We measure both green R&D efficiency and green innovation output efficiency and find that firms in AIDP designated cities achieve significant improvements in both stages of GIE compared with those outside these zones. Mechanism analysis shows that the gains mainly operate through increased AI hardware investment, improved liquidity management, and enhanced ESG engagement, indicating that AIDP facilitates firms’ digital upgrading and responsible innovation. These channels not only strengthen technological and operational capacities but also improve environmental performance and mitigate pollution related health risks. Notably, financial constraints and AI software investment do not serve as significant mediating channels, suggesting that AIDP’s impacts are realized more through tangible technological upgrading and governance improvement rather than short term financial easing. Heterogeneity analysis further reveals that non state-owned enterprises, manufacturing firms, and labor intensive sectors benefit most, suggesting that AI based policies should be tailored to structural characteristics. Finally, we discuss the broader implications of these findings for global AI and sustainability policies, emphasizing how similar initiatives could enhance green innovation and health outcomes beyond China. Our findings make two contributions. First, we expand empirical research on GIE by providing causal evidence of AI policy’s effect, addressing identification challenges that have limited prior work on AI and sustainability. Second, we highlight the public health benefits of AI based green innovation, showing that digital transformation policies can accelerate technological upgrading and environmental sustainability while simultaneously mitigating health risks from pollution exposure. By doing so, the paper contributes to debates on the integration of digital and green transformation and offers transferable insights for policymakers worldwide. This dual contribution provides theoretical insight and practical guidance for aligning digital and environmental policies in emerging economies, emphasizing their joint role in safeguarding population health.

The remainder of the paper is structured as follows. Section 2 outlines the policy background of AIDP and constructs the theoretical framework. Section 3 introduces empirical specifications, data sources, and variable construction. Section 4 presents the baseline regression results and robustness checks. Section 5 examines mechanisms through which AIDP affects GIE. Section 6 explores heterogeneity and robustness extensions. Section 7 concludes with key findings and policy implications.

## 2 Policy background and theoretical framework

### 2.1 Policy background

This section introduces the background of the AIDP policy and explains the theoretical basis for analyzing its impact on firms’ green innovation efficiency. The AIDP were launched by the Chinese government in 2019 as part of a national strategy to promote digital transformation and sustainable industrial development. These pilot zones serve as experimental platforms where AI technologies are integrated into industrial processes, service systems, and urban governance. As of 2022, AIDP has been implemented in a wide range of cities, including Beijing, Shanghai, Nanjing, Hangzhou, Shenzhen, and Chengdu, covering both economically advanced eastern regions and rapidly developing central and western regions. These cities were selected primarily based on their existing digital infrastructure, industrial foundation, and innovation capacity rather than on their green innovation performance. The top-down designation by central authorities rather than local governments or firms themselves provides an exogenous variation in policy exposure, which helps reduce concerns about reverse causality and selection bias. The AIDP policy aims to accelerate AI applications across sectors such as manufacturing, energy, healthcare, and logistics with a specific emphasis on enhancing digital capabilities and supporting low carbon and environmentally friendly innovation. It emphasizes the construction of public data platforms, cross sector collaboration, and the promotion of AI enabled business models. Given the scope and heterogeneity of these pilot zones, they constitute a quasi-experiment to examine the causal effects of AI driven policy interventions. Importantly, the policy coincides with China’s broader national goals of achieving carbon neutrality and green development, linking technological upgrading with environmental governance. Against this backdrop, AIDP provides a relevant institutional shock that allows the investigation of whether state led AI promotion can improve firm level green innovation efficiency both in the upstream R&D stage and the downstream application stage.

Given its broad scope, strong central government backing, and diverse implementation contexts, AIDP provides a rare opportunity to observe how AI policy interventions reshape firms’ innovative systems. Because the policy was not designed around green innovation performance but rather around digital readiness, it introduces an exogenous shift in firms’ technological environment. This enables the examination of how AI promotion can influence GIE through interconnected stages of input, transformation, and output.

### 2.2 Theoretical framework and research hypotheses

AIDP affects green innovation efficiency through a dynamic system of interrelated mechanisms rather than isolated channels. Each mechanism, including financing, internal resources, and operational efficiency, interacts with the others via resource flows, feedback loops, and AI enabled capabilities, forming a coherent framework that facilitates sustained green innovation. Artificial intelligence serves as a central lever that simultaneously enhances multiple mechanisms. AI tools not only streamline R&D and commercialization processes but also improve governance, disclosure, and operational transparency, creating synergistic effects across financing, internal resources, and efficiency channels. Specifically, AI investments improve ESG performance by enabling continuous monitoring, automated disclosure, and compliance support, thereby reducing information asymmetry and increasing investor confidence. They also strengthen liquidity by enhancing process efficiency, reducing waste, and improving financial transparency, which facilitates self-financing of green projects.

Building on this system view, we develop several testable hypotheses to clarify the pathways through which AIDP influences GIE. Artificial intelligence can directly raise both stages of green innovation by changing how firms generate and use knowledge. AI tools enable data driven research, more precise targeting of R&D efforts, and faster iteration through automation and machine learning; these features reduce wasted R&D inputs and make experimental processes more effective (24, 37). At the commercialization stage, AI improves market sensing, shortens product development cycles, and enables continuous monitoring of environmental performance, which helps firms match green products to demand and scale successful innovations (26, 28). Firms located in pilot zones also benefit from shared digital platforms and high-performance computing that raise baseline research productivity (3, 38). That said, we propose the following hypothesis.

*Hypothesis 1*: The establishment of AIDP significantly improves firms’ green R&D efficiency and green innovation output efficiency.

Next, we examine whether AIDP alleviates traditional financing frictions. One fundamental barrier to green innovation is constrained access to external finance. Green projects are often long-term and risky, and they may lack tangible collateral, so capital providers may underwrite them only at high cost or not at all; empirical work shows that financially constrained firms invest less in green R&D and process improvements (39, 40). However, the empirical evidence shows that the Whited Wu (WW) index does not significantly respond to AIDP implementation, suggesting that the policy’s effect on green innovation is not primarily driven by direct alleviation of financial constraints. Instead, the impact seems to operate through alternative pathways such as improved governance, technological upgrading, and ESG performance. At the same time, one must recognize limits: easing external finance does not automatically guarantee better investment decisions if governance is weak or if firms face low absorptive capacity. Through AI enabled decision support, firms can better utilize external funds, reinforcing the interactions between financing and operational efficiency mechanisms.

*Hypothesis 2*: AIDP does not significantly relax firms’ traditional financial constraints; instead, its green innovation effects are transmitted through technology- and governance-related channels.

We then consider the role of internal resource generation, focusing on operating revenue as a potential mechanism. Internally generated funds are an equally important source for green innovation because revenues can be redeployed without the frictions of external finance. Higher operating income signals market acceptance, improves cash flow stability, and increases managerial willingness to invest in long-horizon projects that pay off over time (40). Prior research confirms that sufficient self-financing ability enables firms to overcome cyclical downturns in capital markets and maintain continuous R&D investment, which is particularly crucial for green projects with uncertain payoffs (20, 28). However, the regression results indicate that operating revenue does not significantly increase following AIDP implementation, implying that revenue expansion is not a major mediation channel. A cautionary note is that revenue increases might stem from non-green business lines and not translate automatically into green R&D; the firm’s strategic orientation and incentives therefore matter (37). In our integrated framework, revenue growth functions alongside other mechanisms including liquidity buffers, AI investments, and governance improvements, ensuring that additional sales actually finance environmentally beneficial innovation.

*Hypothesis 3*: AIDP does not significantly affect firms’ operating revenue; the policy’s green innovation effects are more likely mediated by efficiency and governance improvements rather than revenue-driven internal funding.

Moving beyond financing, the fourth hypothesis explores the technological dimension of AI investment. Targeted investments in AI software and hardware are a distinct mechanism because they directly change the firm’s technological capability to carry out and commercialize green innovation. Estimation results show that AIDP significantly increases AI hardware investment, while AI software investment remains insignificant. This indicates that tangible AI capital formation, such as data centers, sensors, and automation hardware, plays a more substantial role than software in enhancing green innovation efficiency. AI capital improves forecasting, reduces uncertainty about demand and process outcomes, and enables fine grained control of energy use and emissions (41). Empirical findings demonstrate that AI adoption significantly enhances green innovation efficiency by optimizing production processes, accelerating green product design, and enabling real time environmental monitoring (26, 42, 43). In addition, AI investments contribute to ESG performance and liquidity improvements, creating reinforcing feedback loops with financing and internal resource mechanisms. AI can also ease financing frictions indirectly by improving firms’ risk profiling and decision support systems, making both managers and outside investors more willing to back green projects (27). At the same time, AI hardware and software require up-front spending and learning; without supporting infrastructure or skilled personnel, such investments may not yield immediate gains. The pilot zones aim to lower these adoption costs through shared resources and knowledge networks, increasing the likelihood that AI investments will translate into sustained green innovation (44). Therefore, we propose the following hypothesis.

*Hypothesis 4*: The AIDP primarily raises AI hardware investment rather than software investment, thereby strengthening firms’ technological capacity and promoting green innovation efficiency.

Given that liquidity underpins firms’ ability to sustain long-term R&D, we next test its mediating role. Liquidity, proxied by current assets, shapes a firm’s ability to absorb shocks and fund long term projects without interruption. Results confirm that AIDP significantly increases firms’ current assets, indicating improved liquidity and stronger short term financial flexibility. Firms with higher liquid buffers can smooth R&D spending through downturns, avoid fire sales of productive assets, and maintain continuous experimentation, conditions especially important for uncertain green projects (40). Prior research indicates that sufficient liquidity enhances resilience against market volatility, allowing firms to sustain green innovation activities despite external shocks (8, 23, 45). AIDP can bolster liquidity indirectly by raising profitability and asset turnover through digital upgrades, and directly by improving access to working capital facilities in pilot regions (44). AI adoption enhances liquidity by improving operational efficiency, reducing production costs, and increasing transparency of financial flows, thereby strengthening firms’ capacity to self-finance green innovation. Critics might argue that excess liquidity can reduce pressure for efficiency or be diverted to nonproductive uses; however, within our system view, liquidity matters because it sits between resource expansion and resource transformation, enabling sustained conversion of inputs into green outputs.

*Hypothesis 5*: The AIDP increases firms’ current assets, thereby enhancing liquidity and facilitating the stable financing of green innovation projects.

Nevertheless, the empirical estimates show that AIDP has no statistically significant effect on total operating costs, suggesting that cost side efficiency improvements are not the dominant pathway. The green economy literature shows that cost efficiency gains often reinforce ecological benefits, creating synergies that strengthen firms’ long term competitive advantage (12, 17, 46). A countervailing risk is that cost reduction can sometimes come at the expense of long term capability building if firms cut R&D budgets to hit short term targets. The AIDP context mitigates this risk by coupling efficiency gains with incentives and infrastructure that channel savings toward sustainable innovation rather than only short term profit maximization (44). We employ the following hypothesis.

*Hypothesis 6*: AIDP does not significantly reduce total operating costs; its influence on green innovation stems mainly from improvements in ESG and liquidity rather than direct cost reduction.

Finally, environmental, social, and governance (ESG) performance creates a reinforcing environment that affects both financing and market opportunities. Consistent with the theoretical framework, ESG performance improves significantly after AIDP implementation, confirming that AI enabled monitoring and compliance systems enhance disclosure quality and sustainability performance. Better ESG scores lower information asymmetry, attract sustainability oriented investors, and can reduce the cost of capital through green credit lines and sustainability linked financing (47, 48). AI tools improve ESG outcomes via continuous environmental monitoring, automated reporting, and compliance management, linking operational efficiency and financing mechanisms with ESG reinforcement. Evidence suggests that AI enabled monitoring and disclosure significantly improve ESG credibility, reducing greenwashing risk and strengthening stakeholder trust (38, 49, 50). AIDP can improve ESG outcomes by facilitating AI based environmental monitoring, enhancing disclosure quality, and supporting compliance with environmental standards (44). In our integrated framework, stronger ESG performance feeds back into easier finance and larger markets, sustaining a virtuous cycle for green innovation (13, 15). That said, we propose the following hypothesis.

*Hypothesis 7*: The AIDP significantly improves firms’ ESG performance, which further reinforces financing capacity and facilitates green innovation.

**Figure 1:**
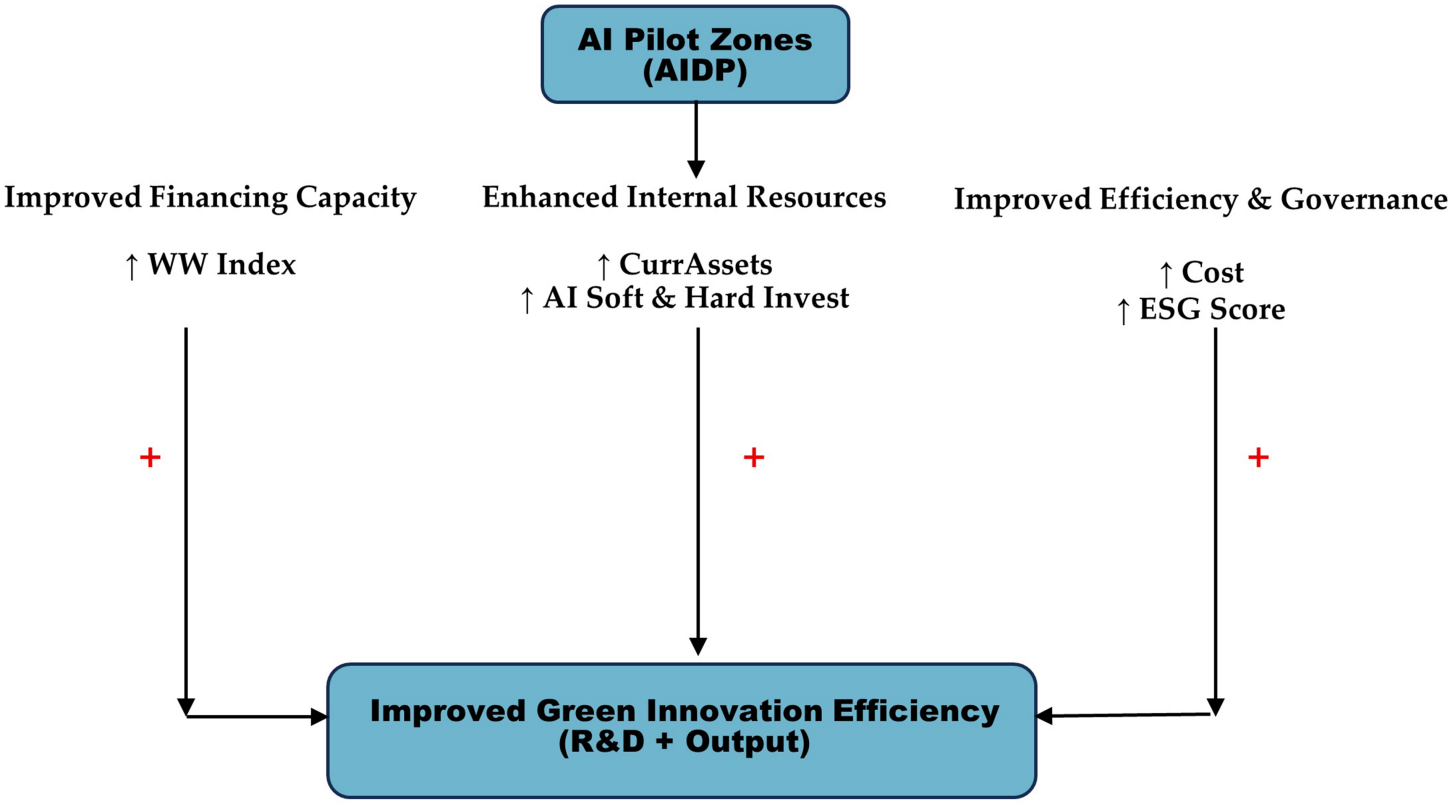
AIDP reshapes firms’ green innovation efficiency.

Figure 1 conceptually depicts the mechanisms through which the Artificial Intelligence Demonstration Policy and firms’ AI investments foster green innovation efficiency. The framework centers on three interlinked channels: financial, resource based, and operational mechanisms. First, the financial mechanism mitigates credit constraints and improves ESG performance, reducing information asymmetry and financing costs. Second, the resource mechanism enhances firms’ internal capital through increased revenue and liquidity, enabling sustained R&D investment. Third, the operational mechanism strengthens innovation efficiency by promoting targeted AI adoption and cost reduction. These channels interact dynamically: AI driven improvements in ESG and liquidity reinforce financial access and internal resource allocation. Overall, the framework illustrates how AIDP reshapes firms’ innovation ecosystems by expanding financing capacity, optimizing internal resources, and enhancing operational efficiency, jointly translating AI driven transformation into sustained green innovation performance.

## 3 Specifications

### 3.1 Variable measurement

#### 3.1.1 Explained variable

This section describes the data, variable definitions, and empirical framework used to measure green innovation efficiency and evaluate the effects of the AIDP policy. Green innovation efficiency (GIE) serves as the core dependent variable in this study. Building on prior literature, we adopt a two-stage framework to evaluate GIE, capturing both the generation and application of green technologies. In the first stage, we measure how effectively R&D inputs such as the number of R&D personnel and R&D expenditure are converted into intermediate outputs, namely green patent applications and grants. The second stage evaluates how these intermediate outputs translate into final outcomes, including sales revenue, energy savings, and pollution reduction. Before conducting the efficiency estimation, all input and output indicators, including R&D expenditure, labor, energy consumption, costs, and pollutant emissions, are standardized to eliminate the influence of scale and unit discrepancies. Continuous variables are further examined for skewness and kurtosis, and extreme observations are winsorized at the 1st and 99th percentiles to reduce the impact of outliers. This two-stage structure highlights the full innovation value chain from knowledge creation to commercialization under environmental constraints, and it emphasizes that improvements in GIE can directly reduce the societal health burden of pollution exposure. Existing studies have rarely examined the causal effects of AI related policies on firm level GIE within such a multidimensional framework, leaving an important gap for empirical research.

To provide a clearer operational definition of GIE, we next explain how green patents, energy use, and pollution indicators are measured in detail. Green patents are identified according to the 2010 WIPO Green Inventory, which maps International Patent Classification (IPC) codes to approximately 200 environmentally relevant topics grouped into seven categories such as renewable energy, transportation, waste management, and energy saving technologies, in line with the UNFCCC guidelines. Firm level green patent data are obtained from the China National Intellectual Property Administration (CNIPA), and industry classification follows the 2017 National Bureau of Statistics standard. Energy consumption is calculated by aggregating firm level usage of water, electricity, coal, natural gas, gasoline, diesel, and centralized heating, and converting these into standard coal equivalents based on national conversion coefficients. To ensure data comparability, energy and cost indicators are deflated to constant prices using the industrial producer price index, and all variables are normalized by firm size or total assets where applicable. Pollution emissions, including COD, ammonia nitrogen, total nitrogen, total phosphorus, SO₂, NOx, and particulate matter, are standardized and combined using the entropy weighting method to construct a comprehensive environmental pollution index. This procedure ensures comparability across firms and accurately reflects both environmental impacts and regional heterogeneity.

To quantify GIE, we employ the super efficiency Slack Based Measure (SBM) model, which addresses the slackness issue inherent in traditional DEA models by incorporating slack variables into the objective function, thus enhancing measurement precision. Importantly, the model accounts for undesirable outputs such as pollution, which is crucial for capturing the environmental dimension of innovation. The general form of the super efficiency SBM model can be found in Appendix.

#### 3.1.2 Key explanatory variable

The key explanatory variable is the AIDP policy, which reflects the influence of digital initiatives on firms’ green innovation efficiency. We define a dummy variable that takes the value of one if firm i is located in city c identified by the State Council as an AIDP pilot zone in year t, and zero otherwise. Since the AIDP was gradually implemented from 2019 onwards, this staggered rollout serves as a quasi-experiment. This setting allows for a multi period DID framework that accounts for time invariant firm characteristics and common shocks, thereby enhancing the credibility of causal identification. The sample covers 2011 to 2022, ensuring an adequate window before and after policy implementation. This variable facilitates identification of whether firms in pilot cities experience greater improvements in green innovation efficiency relative to those in non-pilot cities after the policy was introduced.

#### 3.1.3 Control variables

To address potential omitted variable bias and isolate the AIDP’s effect, we include several widely used firm and region level control variables in the green innovation literature following Mijit et al. (44). Specifically, we control for firm age by including the logarithm of firm age (Age), which may affect a firm’s innovation capacity due to accumulated knowledge and experience. To capture scale effects on innovation, we measure firm size (Size) by the logarithm of total assets. Financial risk and credit constraints are proxied by the leverage ratio (Leverage), computed as total liabilities over total assets. We further include the largest shareholder’s ownership stake (Share) to reflect ownership concentration, which may affect corporate strategies such as green investment. To account for firm level productivity differences, we control for total factor productivity (TFP). Finally, to capture regional digital infrastructure and its enabling role in green innovation, we include the provincial digitalization index (Digital). These control variables help ensure that our results are not confounded by other firm level characteristics or regional development trends that might also affect green innovation outcomes (26, 45).

#### 3.1.4 Data sources and descriptive statistics

The primary firm level data for this study are drawn from the China Stock Market and Accounting Research (CSMAR) database, which offers detailed financial and corporate information for China’s publicly listed companies. To supplement this, we also collect data from firms’ annual reports, corporate social responsibility (CSR) reports, and official company websites. These sources provide comprehensive information on firms’ R&D activities, financial performance, and environmental disclosures, which are essential for constructing our measures of green innovation efficiency and control variables. Information on the Artificial Intelligence Innovation and Development Pilot Zones (AIDP) comes from official announcements by the State Council of China, as published on the central government website. This source allows us to accurately identify the timing and location of pilot zone designations for our DID analysis.

To ensure robustness and comparability, we merge firm data from CSMAR, CNIPA, and AIDP announcements by firm code and fiscal year to form an unbalanced panel dataset. All monetary variables are deflated to 2015 constant prices using the industrial producer price index. Variables reflecting firm innovation inputs, such as R&D investment, R&D personnel, and operating costs, are standardized by total assets or number of employees to control for firm scale heterogeneity. Similarly, energy consumption and environmental expenditure variables are normalized by operating revenue to ensure comparability across industries and firm sizes.

Continuous variables are examined for skewness, kurtosis, and extreme values, with outliers winsorized at the 1st and 99th percentiles. Variables exhibiting significant skewness, including Revenue, CurrAssets, Cost, AISoftInvest, and AIHardInvest, are log transformed to mitigate heteroskedasticity and improve comparability across firms. Green innovation efficiency scores calculated using the two stage DEA SBM model are normalized to the [0,1] range to ensure consistent interpretation. Observations with excessive missingness are excluded, while limited missing data are imputed using conditional mean methods within industry year cells. The descriptive statistics in Table 1 summarize all key variables, confirming that they are suitably distributed for DID regression with no distortions that violate linear modeling assumptions.

**Table 1:** Summary statistics.

Variables	Definition	*N*	Mean	SD	Min	Max	Skewness	Kurtosis
Explanatory Variable								
AIDP	Dummy variable	23,682	0.176	0.381	0	1	1.703	3.903
Explained variables								
GrdE	Green R&D efficiency	23,682	0.575	0.200	0.134	1	0.248	2.351
GopE	Green innovation output efficiency	23,682	0.574	0.199	0.134	1	0.260	2.384
Control variables								
Age	Logarithm of firm age	23,682	2.189	0.761	0.693	3.496	−0.399	2.086
Size	Firm size	23,682	22.321	1.344	17.804	28.636	0.933	4.185
Leverage	Total liabilities / total assets	23,682	0.425	0.204	0.007	1.718	0.248	2.496
Share	Shareholding ratio of the largest shareholder	23,682	34.471	14.814	0.286	89.986	0.537	2.912
TFP	Total factor productivity	23,682	5.590	0.778	2.723	9.015	0.504	3.363
Digital	Provincial digitalization level index	23,682	340.103	113.223	7.580	467.172	−1.237	3.711
Mechanism variables								
WW	Whited–Wu index for financial constraint	23,682	−1.035	0.536	−7.075	−0.699	−103.244	11873.56
Revenue	Operating revenue (in million RMB)	23,682	12913.950	91761.660	0	3.318	22.867	622.968
AISoftInvest	AI software investment (in million RMB)	18,667	18.299	77.732	0	29.287	16.442	422.076
AIHardInvest	AI hardware investment (in million RMB)	9,676	58.726	216.930	0	41.340	9.481	118.745
CurrAssets	Current assets (in million RMB)	23,682	4459.589	15538.100	0	44.109	14.922	296.322
Cost	Operating cost (in million RMB)	23,682	12152.840	87993.570	0	3.232	22.942	625.449
ESG	Average ESG score	23,682	4.107	0.950	1	7.750	−0.321	3.456

### 3.2 Baseline model

Our study examines the effect of the AIDP policy on firm level green innovation efficiency in China from 2011 to 2022. Leveraging the staggered implementation of AIDP across different cities as a quasi-experiment, we employ a difference in differences (DID) approach (51). This method compares firms located in designated pilot cities with those in non-pilot cities before and after the policy rollout. The DID framework is particularly suitable because it controls for unobservable, time invariant firm characteristics and common shocks over time, thereby mitigating endogeneity concerns arising from policy placement and firm specific innovation capabilities (12, 13). Moreover, the DID approach has been widely applied in recent empirical studies evaluating place based policies and innovation reforms, particularly when treatment timing varies across units, which provides robust causal inference (8, 52). The baseline model is specified in Equation 1 as follows:


$$Y_{\mathrm{ict}}=\beta_{0}+\beta_{1}\mathrm{AID}P_{\mathrm{ict}}+\gamma X_{\mathrm{it}}+\delta_{\mathrm{jt}}+\rho_{\mathrm{et}}+\mu_{i}+\theta_{t}+\varepsilon_{\mathrm{ict}}$$ (1)

Where the dependent variable $Y_{\mathrm{ict}}$ ​ denotes green innovation efficiency for firm i in city c at year t comprising two dimensions: green R&D efficiency ( GrdE ) and green innovation output efficiency ( GopE ). The key explanatory variable $\mathrm{AID}P_{\mathrm{ict}}$ is a binary indicator equal to one if firm i is in a city ccc covered by the *AIDP* policy in year t , and zero otherwise. $X_{\mathrm{it}}$ represents a vector of firm-level control variables. To address confounding factors, the model incorporates province by year fixed effects ( $\delta_{\mathrm{jt}}$ ) capturing regional shocks and policy heterogeneity, industry by year fixed effects ( $\rho_{\mathrm{et}}$ ) controlling for sector specific trends, firm fixed effects ( $\mu_{i}$ ) absorbing time invariant firm heterogeneity, and year fixed effects ( $\theta_{t}$ ) accounting for nationwide macroeconomic fluctuations. Standard errors are clustered at the city level, in line with common practice in policy evaluation research (16, 42). While the baseline DID model establishes the overall treatment effect, understanding how the AIDP policy operates requires further exploration of the underlying channels. The next section therefore develops a mechanism analysis framework.

### 3.3 Mechanism model

To uncover the channels through which the AIDP policy influences firm level green innovation efficiency, we extend the baseline DID model by including interaction terms between the treatment indicator and potential mechanism variables. We adopt a sequential mediation framework in which the AIDP policy affects intermediate firm level mechanisms that in turn influence green innovation efficiency. We examine six critical mechanisms through which the AIDP may enhance green innovation: financial constraints, internal resources, AI investment, liquidity, operating cost efficiency, and ESG performance. In the first stage, we estimate the impact of AIDP on each mechanism variable using a DID specification given in Equation 2:


$$M_{\mathrm{it}}=\alpha +\beta_{1}(\mathrm{AID}P_{\mathrm{ct}}\times \mathrm{Pos}t_{t})+\gamma X_{\mathrm{it}}+\mu_{i}+\lambda_{t}+\epsilon_{\mathrm{it}}$$ (2)

Where $M_{\mathrm{it}}$ represents the mechanism (e.g., WW index, AI investment, liquidity, cost, ESG), and other notations are as defined previously. In the second stage, we link these mechanisms to firms’ green innovation efficiency to verify the mediation pathway, as specified in Equation 3:


$$Y_{\mathrm{ict}}=\alpha +\beta_{2}(\mathrm{AID}P_{\mathrm{ct}}\times \mathrm{Pos}t_{t})+\delta M_{\mathrm{it}}+\gamma X_{\mathrm{it}}+\mu_{i}+\lambda_{t}+\epsilon_{\mathrm{it}}$$ (3)

Here, $Y_{\mathrm{ict}}$ ​​ remains as defined in the baseline model, $\mathrm{AID}P_{\mathrm{ict}}$ ​ is the policy treatment dummy, and $M_{\mathrm{it}}$ ​ denotes a time varying firm level mechanism variable. We investigate six critical mechanisms through which the *AIDP* may enhance green innovation. Financial constraints are measured using the Whited Wu index, as tighter external financing restricts investment in uncertain long term green innovation projects, while a lower index value indicates relaxed constraints that foster eco innovation. Operating revenue (Revenue) captures internal funding capacity, where higher revenue reflects stronger profitability and greater resources for green innovation independent of external capital. Investments in AI software ( AISoftInvest ) and AI hardware ( AIHardInvest ) represent distinct mechanisms because AI deployment enhances operational efficiency, reduces uncertainty, and improves risk management, thereby easing financing frictions and directing managerial attention toward sustainable innovation. Current assets (CurrAssets) indicate a firm’s liquidity buffer that helps maintain steady R&D funding even under credit tightening. Operating cost (Cost) reflects resource efficiency, where lower costs allow firms to reallocate resources to green technology development. Finally, ESG performance captures environmental responsibility and governance quality, improving access to green finance and reducing capital costs. Collectively, these mechanisms explain how AIDP promotes green innovation by strengthening financing conditions, optimizing internal resource allocation, and improving sustainability governance.

## 4 Results

### 4.1 Baseline regression results

This section reports the main empirical findings and shows how the AIDP policy improves firms’ green innovation efficiency across different dimensions. The DID estimation reveals a consistent and positive impact of the AI Pilot Zone policy on firms’ green innovation efficiency. Following the empirical strategy outlined in Section 3.1, we employ a two way fixed effect DID model with standard errors clustered at the city level across 260 cities to estimate the causal effect of the AIDP policy on firms’ green innovation efficiency during 2011–2022. Table 2 reports the estimation results for two dimensions of green innovation efficiency: green R&D efficiency ( GrdE ) and green innovation output efficiency ( GopE ).

**Table 2:** Baseline regression results.

Variables	(1)	(2)	(3)	(4)	(5)	(6)	(7)	(8)
	*GrdE*	*GrdE*	*GrdE*	*GrdE*	*GopE*	*GopE*	*GopE*	*GopE*
DID	0.163***	0.054***	0.013***	0.017***	0.162***	0.053***	0.017***	0.014**
	(0.003)	(0.003)	(0.005)	(0.006)	(0.003)	(0.003)	(0.006)	(0.006)
Age		0.003**	0.008**	0.007**		0.006***	0.011***	0.009***
		(0.001)	(0.004)	(0.004)		(0.001)	(0.003)	(0.003)
Size		−0.005***	−0.005	−0.005		−0.010***	−0.007**	−0.008**
		(0.001)	(0.003)	(0.003)		(0.001)	(0.003)	(0.004)
Leverage		−0.019***	−0.015	−0.017		−0.011*	−0.012	−0.014
		(0.006)	(0.013)	(0.013)		(0.006)	(0.010)	(0.011)
Share		−0.000	−0.000	−0.000		0.000	0.000	−0.000
		(0.000)	(0.000)	(0.000)		(0.000)	(0.000)	(0.000)
TFP		0.014***	0.008**	0.008**		0.018***	0.008**	0.010***
		(0.002)	(0.003)	(0.004)		(0.002)	(0.004)	(0.004)
Digital		0.001***	0.000	0.001		0.001***	0.000	0.001
		(0.000)	(0.000)	(0.001)		(0.000)	(0.000)	(0.001)
Number of clusters (City)		260						
Observations	23,682	23,682	23,682	23,682	23,682	23,682	23,682	23,682
R-squared	0.097	0.359	0.551	0.561	0.097	0.361	0.546	0.557
Firm FE	No	No	Yes	Yes	No	No	Yes	Yes
Year FE	No	No	Yes	Yes	No	No	Yes	Yes
Prov × Year FE	No	No	No	Yes	No	No	No	Yes
Ind × Year FE	No	No	No	Yes	No	No	No	Yes

This table reports the baseline DID estimates of the AI Pilot Zone Policy on firms’ green R&D efficiency (GrdE) and green innovation output performance (GopE). The main regressor, DID, equals one for firms in pilot cities after policy implementation. All regressions are estimated via reghdfe, with standard errors clustered at the city level. Firm fixed effects account for time-invariant characteristics; year fixed effects capture nationwide shocks; province-by-year fixed effects absorb regional variations over time; and industry-by-year fixed effects control for sector-specific temporal trends. ***, **, and * indicate significance at the 1, 5, and 10% levels.

In Column (1), the baseline regression without any control variables or fixed effects shows that the implementation of AIDP policies significantly improves firms’ green R&D efficiency, with a DID coefficient of 0.163 (*p* < 0.01). This indicates that, on average, firms located in AIDP cities improved their GrdE by 16.3% more than those in non-pilot cities after the policy implementation. A similar effect is observed for GopE in Column (5), with a coefficient of 0.162 (*p* < 0.01), suggesting that AI policy not only fosters green R&D activities but also enhances the transformation of these efforts into green outputs. When firm level control variables are included in Columns (2) and (6), the magnitude of the coefficients on the DID variable decreases to 0.054 for GrdE and 0.053 for GopE , though both remain highly significant. This attenuation suggests that part of the raw effect may be explained by firm characteristics such as firm age, size, leverage, ownership structure, TFP, and digitalization level.

Columns (3) and (7) incorporate firm and year fixed effects to control for unobserved firm heterogeneity and nationwide shocks. The DID coefficients remain positive and significant at the 1 percent level ( GrdE : 0.013; GopE : 0.017), confirming a stable policy effect. Columns (4) and (8) adopt the most rigorous specification by additionally including province by year and industry by year fixed effects to capture regional and sectoral dynamics. The DID estimates remain significant ( GrdE : 0.017, *p* < 0.01; GopE : 0.014, *p* < 0.05), confirming the robustness of the baseline results.

In additional, to further strengthen the causal interpretation of our baseline results, we incorporate city-specific linear trends into the DID specification to account for potential unobserved, time varying factors that evolve differently across cities. Specifically, we augment the model by interacting a city identifier with a continuous year trend term ( c.citytrend#i.city_id ), which flexibly captures heterogeneous growth trajectories in local economic structures, innovation environments, or policy intensities. After establishing the baseline relationship, we next test whether these results hold when accounting for potential city-level unobserved heterogeneity.

The estimation results, presented in Table 3, indicate that including city specific trends does not alter the qualitative conclusions. The DID coefficients remain positive and statistically significant for both green R&D efficiency (GrdE) and green innovation output efficiency (GopE), confirming that the observed effects are not driven by pre-existing city-level trends. These results reinforce the robustness of the positive influence of the AI Pilot Zone policy on firms’ green innovation efficiency.

**Table 3:** City specific linear trends and never treated controls.

Variables	(1)	(2)	(3)	(4)
	*GrdE*	*GopE*	*GrdE*	*GopE*
DID	0.017**	0.020***	0.063***	0.073***
	(0.008)	(0.007)	(0.007)	(0.007)
Age	0.005**	0.009***	0.001	0.003
	(0.002)	(0.002)	(0.004)	(0.004)
Size	−0.001	−0.005***	−0.003	−0.013***
	(0.001)	(0.001)	(0.003)	(0.003)
Leverage	−0.016**	−0.008	0.007	0.012
	(0.007)	(0.006)	(0.016)	(0.016)
Share	0.000	0.000	0.000	0.000
	(0.000)	(0.000)	(0.000)	(0.000)
TFP	0.003	0.006***	0.006	0.014***
	(0.002)	(0.002)	(0.005)	(0.005)
Digital	−0.000	0.001	0.001***	0.001***
	(0.001)	(0.001)	(0.000)	(0.000)
*City-specific trends (c.citytrend#i.city_id)*	Included	Included		
*Never-Treated Controls*			Included	Included
Number of clusters (City)	260			
Observations	23,682	23,682	3,705	3,705
R-squared	0.097	0.097	0.374	0.362
Firm FE	Yes	Yes	Yes	Yes
Year FE	Yes	Yes	Yes	Yes
Prov × Year FE	Yes	Yes	Yes	Yes
Ind × Year FE	Yes	Yes	Yes	Yes

All regressions are estimated via reghdfe, with standard errors clustered at the city level. ***, **, and * indicate significance at the 1, 5, and 10% levels.

We further enhance our baseline analysis by (i) incorporating city specific linear trends to capture potential heterogeneous time dynamics across cities, and (ii) conducting an additional robustness check that restricts the sample to later-treated cities as the treatment group and never treated cities as the control group. Columns (1)–(2) of Table 3 present the baseline results with city specific trends, while Columns (12)–(13) display the robustness results based on the restricted subsample. Across all specifications, the estimated coefficients on the DID variable remain positive and highly significant for both green R&D efficiency and green innovation output efficiency. These findings confirm that the observed positive effects of the AI Pilot Zone policy on firm’ green innovation are robust and not driven by sample selection or unobserved city specific trends.

### 4.2 Parallel trend test

**Figure 2:**
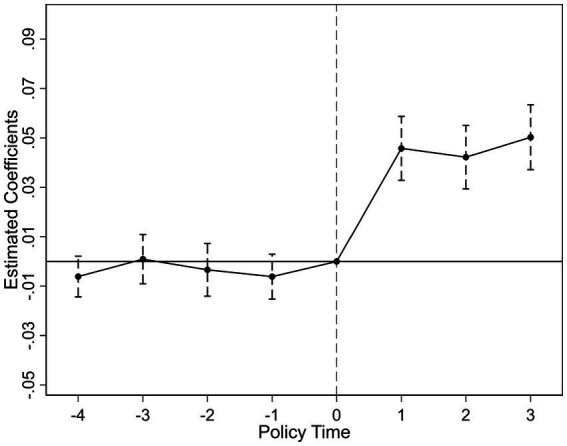
Parallel trend test (green R&D efficiency).

**Figure 3:**
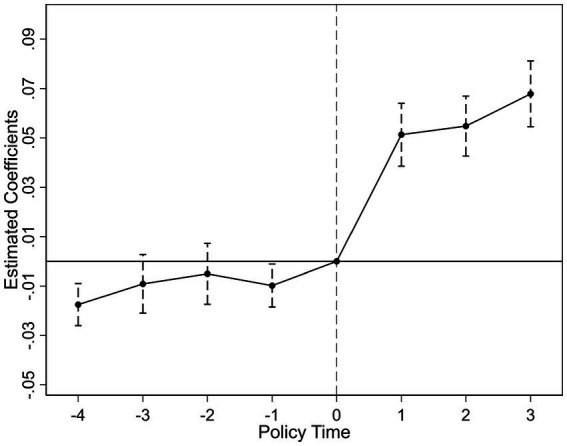
Parallel trend test (green innovation output efficiency).

To validate the DID identification, we conduct parallel trend tests and dynamic analysis. Figures 2, 3 present the visual evidence for green R&D efficiency ( GrdE ) and green innovation output efficiency ( GopE ), respectively. Both figures consistently demonstrate that the 95% confidence intervals for all pre-treatment coefficients encompass zero. Crucially, the point estimates exhibit no systematic divergence or trend divergence in the years preceding the policy shock. For instance, in Figure 2, the pre-treatment coefficients fluctuate marginally between −0.05 and 0.03, with no statistically significant deviations. Similarly, Figure 3 shows pretreatment estimates bounded within [−0.04, 0.02], all statistically insignificant at conventional levels. This pattern holds robustly across both efficiency metrics, indicating that treatment and control groups followed statistically parallel paths prior to 2019.

In the post-treatment period, we observe a statistically significant upward shift in both outcomes. For GrdE rise monotonically from 0.12 to 0.24, while GopE coefficients increase from 0.08 to 0.19. This delayed positive response aligns with theoretical expectations, as the adoption of artificial intelligence technologies and the green innovation process often involve time lags before efficiency gains become evident. Furthermore, the parallel trend assumption remains valid after controlling for province by year and industry by year effects, firm level heterogeneity, and macroeconomic fluctuations, as specified in our fixed effect structure.

### 4.3 Robustness checks

#### 4.3.1 Placebo tests

We conduct a series of robustness checks to ensure the reliability of our baseline results and address potential concerns related to model specification, sample heterogeneity, and endogeneity. These tests collectively assess whether the observed effects of the Artificial Intelligence Development Policy (AIDP) on green innovation efficiency remain consistent under alternative assumptions and empirical designs.

**Figure 4:**
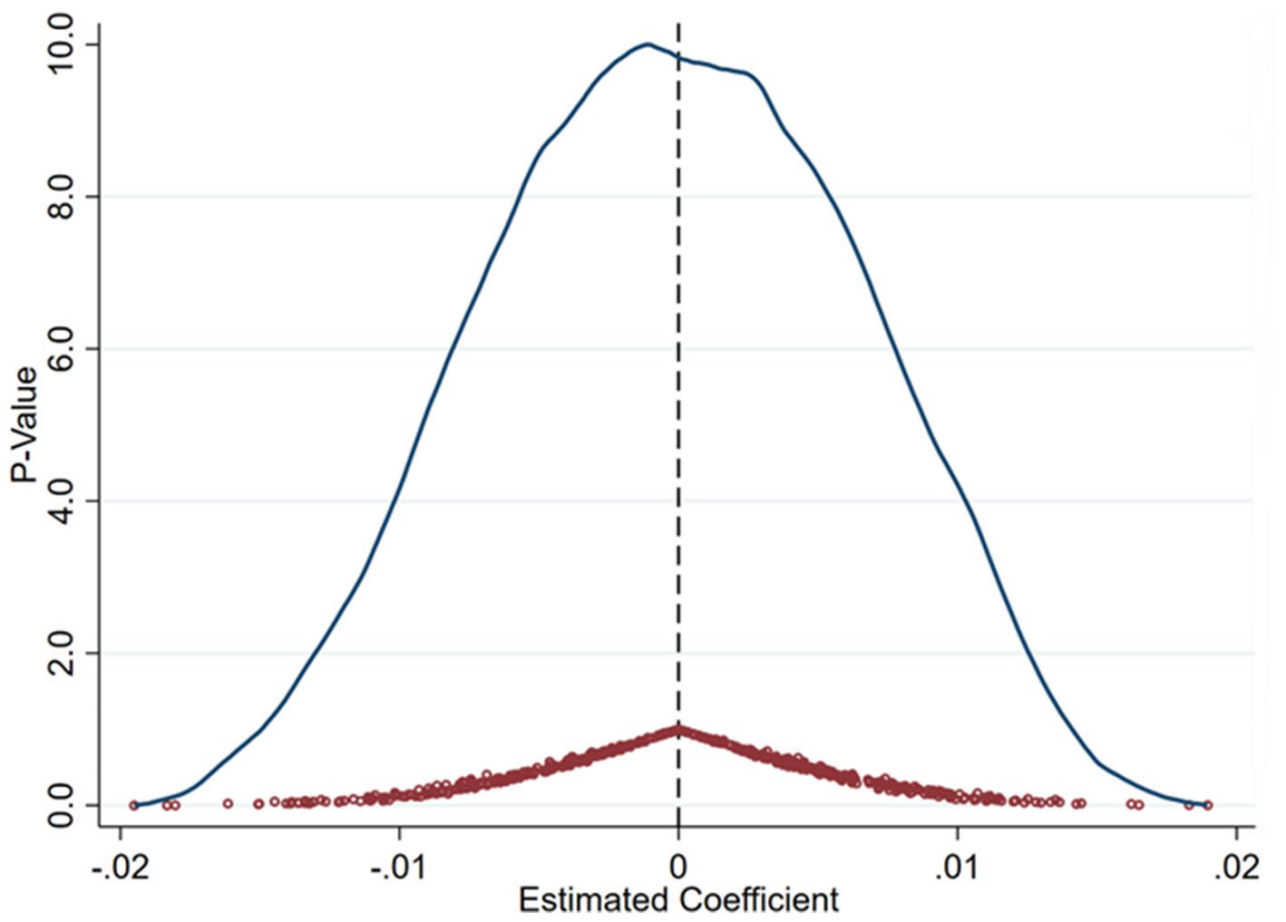
Placebo test (green R&D efficiency).

**Figure 5:**
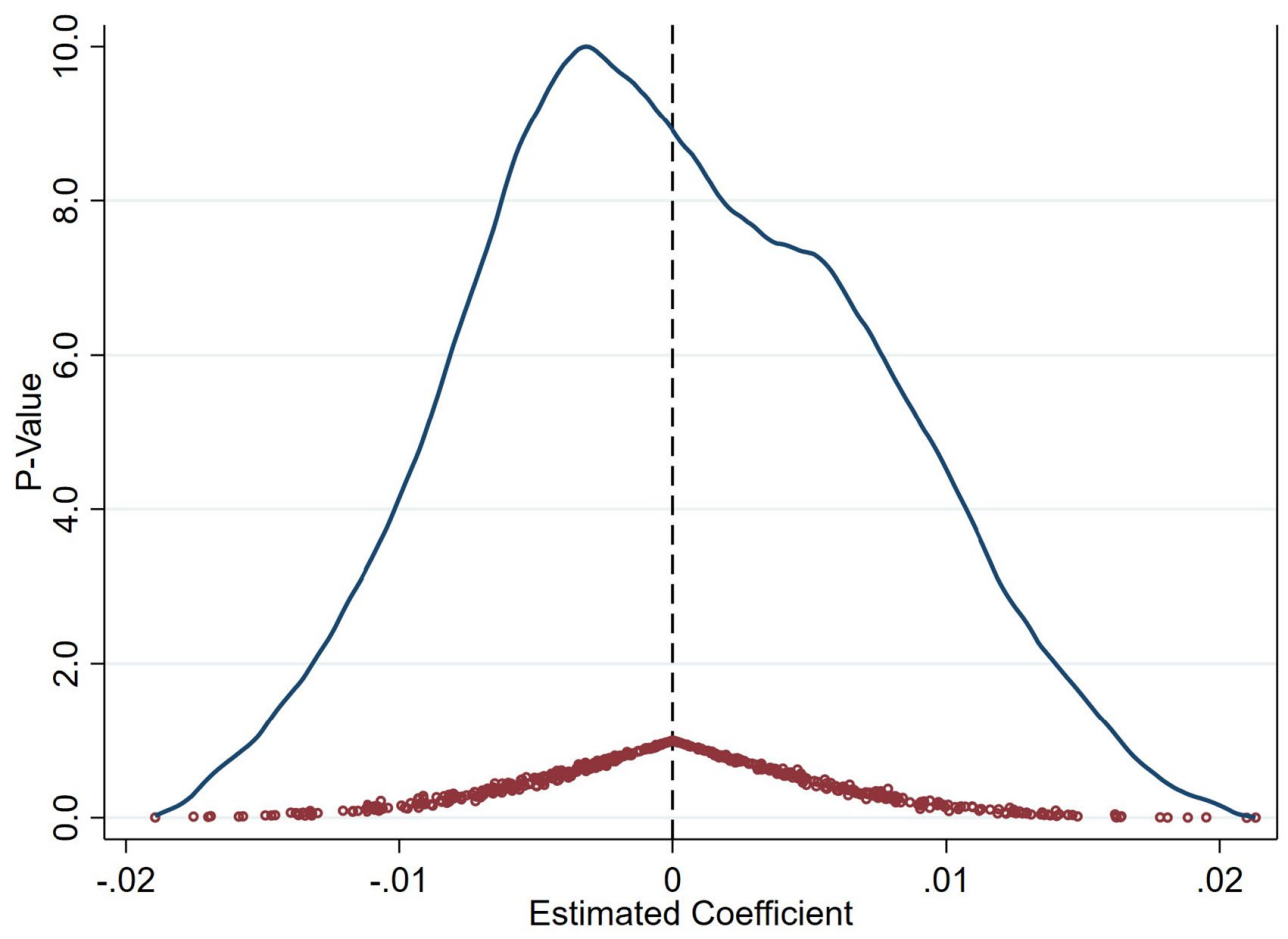
Placebo test (green innovation output efficiency).

A key concern is that the estimated treatment effects might reflect pre-existing trends or anticipatory responses rather than the true impact of policy implementation. To rule out this possibility, we conduct placebo tests by artificially assigning policy implementation years prior to the actual launch of the AI Pilot Zones in 2019. Specifically, we assign pseudo treatment years to periods prior to the actual policy implementation. In our baseline setting, the AI Innovation and Development Pilot Zones were officially launched in 2019. To construct placebos, we artificially shift the treatment year backward and re-estimate the model using 2018, 2017, and 2016 as “placebo” policy years. If the DID coefficients under these placebo years are statistically insignificant, it would suggest that no anticipatory or confounding policy effects existed prior to 2019, thus lending credibility to the parallel trends assumption. We report full regression outputs in Table 4, including pre-treatment mean outcomes, standard deviations, and *p*-values, to provide transparency and allow replication. All placebo regressions are estimated with standard errors clustered at the city level (260 clusters). These results indicate that the pre-policy periods did not experience similar changes as observed after the true policy implementation, strengthening our confidence that the estimated treatment effects are indeed driven by the AI policy intervention rather than unobserved trends. The overall distributional evidence further supports this conclusion. Figures 4, 5 illustrate the distributions of estimated coefficients and *p*-values obtained from multiple placebo iterations, where treatment assignments are randomly permuted across cities and years. The placebo estimates cluster tightly around zero, while the actual estimated coefficient lies at the extreme tail of the distribution. Such a pattern provides strong empirical support that the identified policy effects are not due to random variation, affirming the causal impact of the AI Pilot Zone policy on green innovation efficiency. Building upon these placebo results, we next examine whether the dynamic evolution of treatment effects over time remains consistent when accounting for staggered policy implementation.

**Table 4:** Placebo test.

Variables	(1)	(2)	(3)	(4)	(5)	(6)
	*GrdE*	*GopE*	*GrdE*	*GopE*	*GrdE*	*GopE*
$\mathrm{DID}\_\text{placeb}o_{t-1}$	0.006	0.003				
	(0.007)	(0.007)				
$\mathrm{DID}\_\text{placeb}o_{t-2}$			0.006	0.003		
			(0.007)	(0.007)		
$\mathrm{DID}\_\text{placeb}o_{t-3}$					0.006	0.003
					(0.007)	(0.007)
Number of clusters (City)	260					
Observations	23,682	23,682	23,682	23,682	23,682	23,682
R-squared	0.564	0.558	0.564	0.558	0.564	0.558
Firm FE	Yes	Yes	Yes	Yes	Yes	Yes
Year FE	Yes	Yes	Yes	Yes	Yes	Yes
Prov × Year FE	Yes	Yes	Yes	Yes	Yes	Yes
Ind × Year FE	Yes	Yes	Yes	Yes	Yes	Yes
Controls	Yes	Yes	Yes	Yes	Yes	Yes

The table presents placebo regressions that test the validity of the DID common trend assumption. Placebo policy dummies are created for pre-intervention years, and the baseline model is re-estimated. Columns (1)–(2) assign 2018 (t-1) as the placebo year, Columns (3)–(4) assign 2017 (t-2), and Columns (5)–(6) assign 2016 (t-3). The actual AI Pilot Zone policy commenced in 2019. Standard errors are clustered at the city level (260 clusters). ***, **, and * denote significance at the 1, 5, and 10% thresholds.

#### 4.3.2 Dynamic treatment effects under staggered policy implementation

The AI Pilot Zone policy was introduced at different times across cities, which may introduce potential biases when using conventional two-way fixed effects estimators in the presence of heterogeneous treatment timing. This issue is addressed by applying the estimator proposed by Callaway and Santanna (53), which extends the difference in differences framework to accommodate multiple time periods with staggered adoption. The method identifies group and time-specific average treatment effects using not-yet-treated units as controls, thereby avoiding contamination from already treated groups and ensuring valid inference under heterogeneous effects. For robustness, the results from the standard two-way fixed effects model are reported together with those from the Sun and Abraham estimator. The consistency in the direction and magnitude of coefficients across the two estimators confirms that the results are not sensitive to alternative estimation strategies. Furthermore, the pre-treatment coefficients are jointly insignificant based on the pre-period F-test (*p* > 0.10), supporting the validity of the parallel trends assumption.

**Figure 6:**
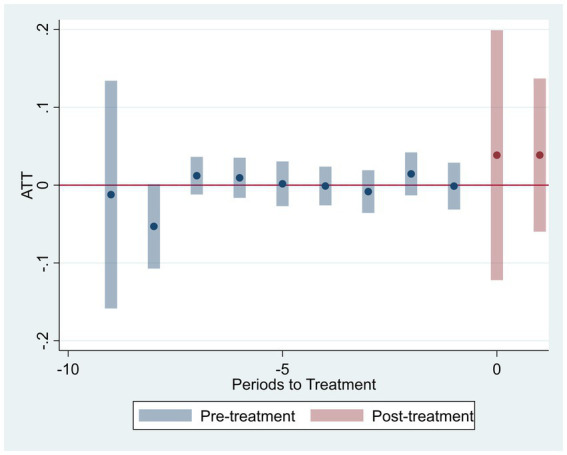
Dynamic effects on green R&D efficiency.

**Figure 7:**
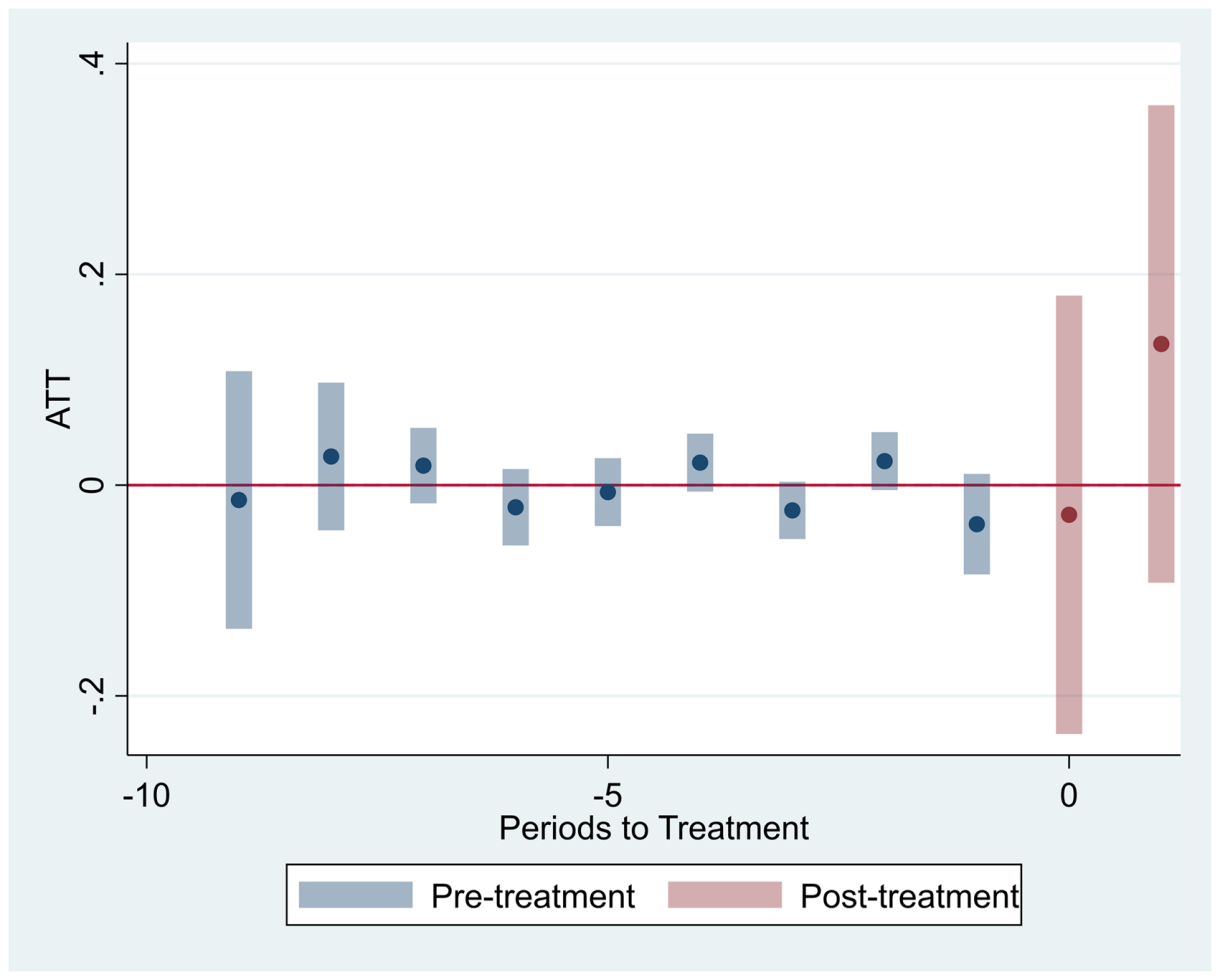
Dynamic effects on green innovation output efficiency.

Figures 6, 7 display the dynamic treatment effects on firm-level green R&D efficiency (GrdE) and green innovation output efficiency (GopE). The estimated coefficients before the policy implementation fluctuate closely around zero and remain statistically insignificant, suggesting no systematic pre-trend differences between treated and control groups. After policy implementation, the coefficients generally become positive, though with wider confidence intervals, indicating that the AI Pilot Zone policy progressively enhanced green innovation efficiency as implementation deepened.

Overall, the dynamic evolution of treatment effects aligns well with the baseline results. The absence of notable pre-policy deviations reinforces the credibility of the identification strategy, while the gradually increasing post-treatment coefficients imply that the benefits of the AI Pilot Zone policy on green innovation efficiency emerge over time. These findings indicate that the core conclusions remain stable under estimation methods that account for staggered treatment timing and heterogeneous policy effects. Having verified that the estimated impacts are not artifacts of treatment timing or pre-trend violations, the following section further strengthens causal inference by addressing potential selection bias through a Propensity Score Matching Difference in Differences (PSM-DID) approach.

#### 4.3.3 Propensity score matching difference-in-differences

The Difference in Differences (DID) model effectively controls for unobserved firm characteristics that do not vary over time. However, treated and control firms may still differ systematically in observable dimensions, which could generate selection bias. To strengthen the credibility of the causal inference, this section applies the Propensity Score Matching Difference in Differences (PSM DID) approach. The method integrates matching and DID by balancing observable attributes between treated and control firms before estimating treatment effects, thus mitigating potential bias from non-comparable samples. Treated firms are matched with comparable untreated firms based on pre-treatment firm characteristics, including firm age, size, leverage, ownership concentration, total factor productivity (TFP), and the provincial digitalization index. Matching is performed using one-to-five nearest neighbor matching with a caliper of 0.05, and all observations are restricted to the common support region to ensure comparability between the two groups.

**Figure 8:**
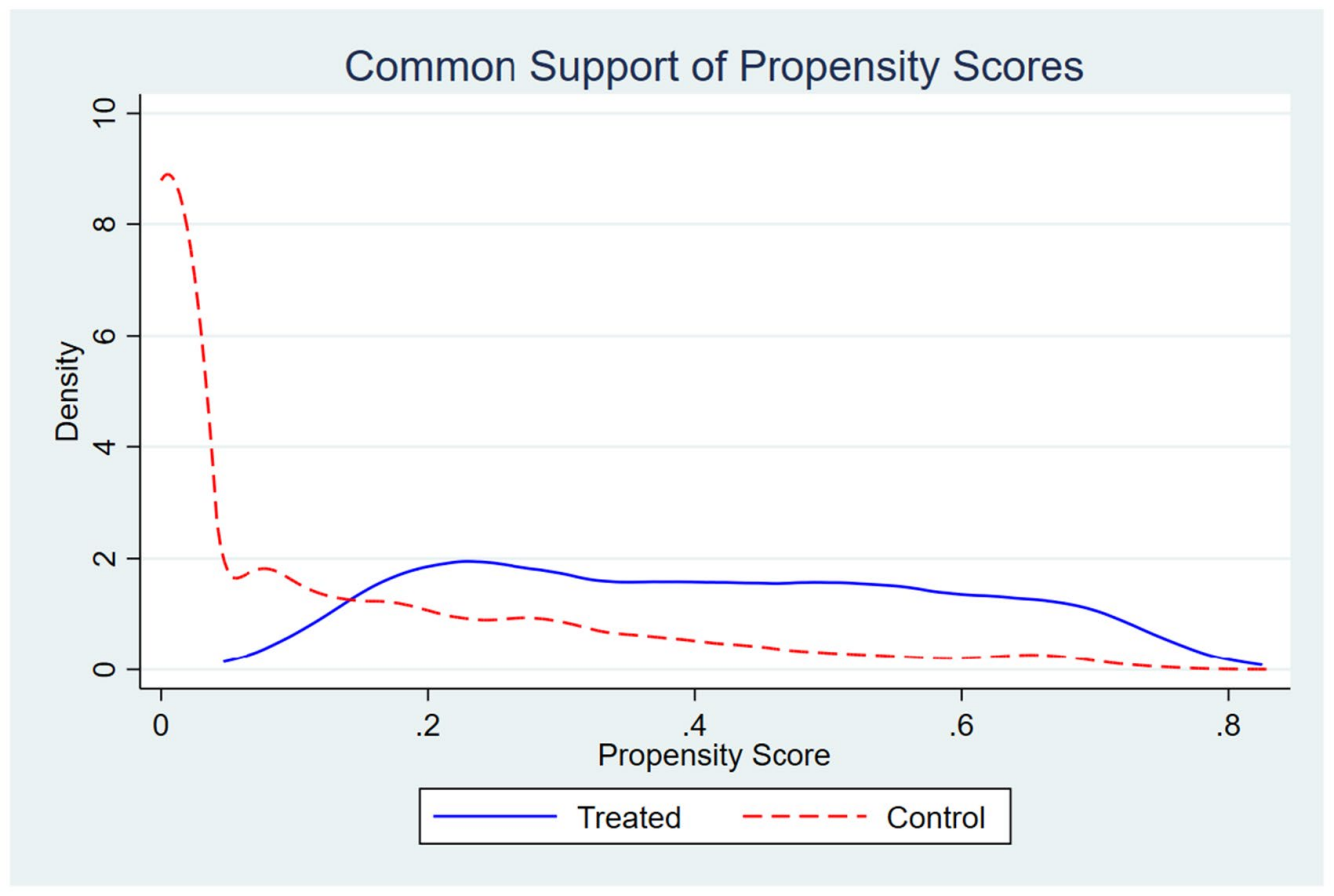
Covariate balance before and after matching.

The common support plot (Figure 8) displays the overlap of the estimated propensity score distributions for treated and control firms, confirming that sufficient overlap exists to ensure credible matching. After matching, 4,160 treated firms are successfully paired with 19,514 control firms, as reported in Supplementary Table 1 and Figure 1. This indicates that most treated observations find suitable counterparts within the support region, thereby satisfying the overlap condition required for PSM DID estimation.

**Figure 9:**
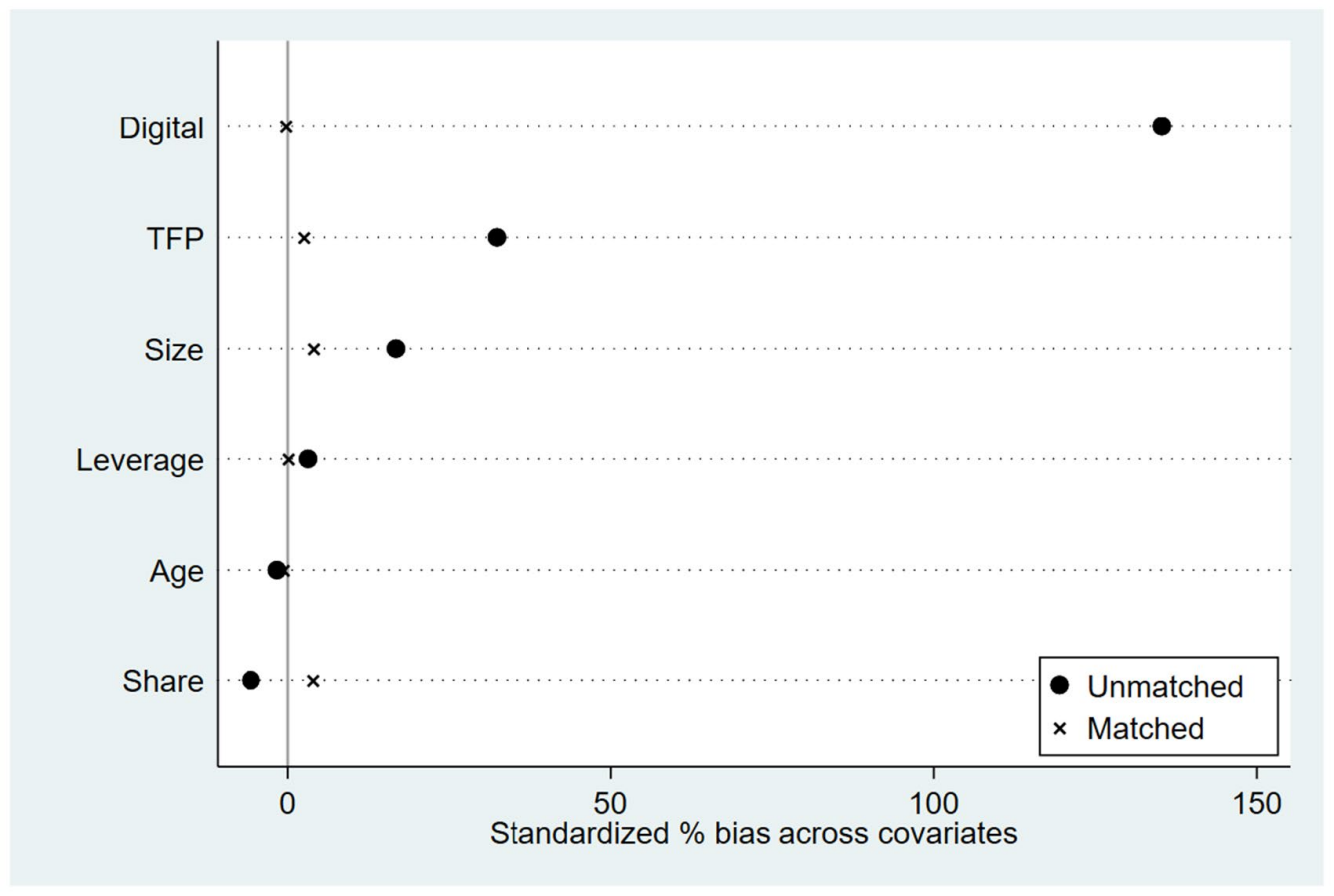
PSM balance test. The figure shows standardized mean differences of covariates before and after matching. Substantial reductions in bias indicate good covariate balance between treated and control firms.

The quality of matching is evaluated through covariate balance diagnostics. Figure 9 presents standardized mean differences (SMDs) of all covariates before and after matching. The results show that the bias for all covariates is substantially reduced, with most differences approaching zero, indicating a high degree of balance between treated and control firms after matching. Table 5 further reports SMDs before and after matching, and Table 6 summarizes the matched sample composition and key summary statistics, all confirming that the PSM procedure effectively mitigates observable selection bias.

**Table 5:** Covariate balance before and after matching.

Variable	Treated (Unmatched)	Control (Unmatched)	%Bias (Unmatched)	Treated (Matched)	Control (Matched)	%Bias (Matched)
Age	2.1791	2.192	−1.7	2.1791	2.1841	−0.6
Size	22.514	22.28	16.7	22.514	22.458	4.0
Leverage	0.43017	0.42381	3.1	0.43017	0.42995	0.1
Share	33.766	34.62	−5.8	33.766	33.189	3.9
TFP	5.797	5.5454	32.3	5.797	5.7774	2.5
Digital	432.39	320.43	135.3	432.39	432.61	−0.3

**Table 6:** Sample characteristics before and after.

Matching sample	Treated Obs	Control Obs	Ps R^2^	LR chi^2^	Mean Bias	%Var
Unmatched	2,345	21,329	0.332	7308.320	32.500	67
Matched	2,345	2,390	0.001	7.530	1.900	83

Table 7 reports the PSM DID estimation results for green innovation efficiency. Across model specifications with and without firm, year, province by year, and industry by year fixed effects, the DID coefficients remain positive and statistically significant. The AI Pilot Zone policy increases GrdE by 0.018 to 0.025 and GopE by 0.016 to 0.027, all significant at the 1 percent level. These findings imply that the positive effects of the policy are not the result of non-random sample selection but represent a stable causal relationship. The consistency between the PSM DID and baseline DID results reinforces the reliability of the identification strategy and confirms that AI policy interventions significantly improve firm-level green innovation efficiency. All standard errors are clustered at the city level (260 cities: 17 treated, 243 control), and the results remain qualitatively unchanged. Since the PSM-DID estimates are consistent with the baseline findings, we proceed to test whether these results are robust to different inference procedures and clustered standard error corrections.

**Table 7:** Sample characteristics before and after matching.

Variables	(1)	(2)	(3)	(4)
	*GrdE*	*GrdE*	*GopE*	*GopE*
DID	0.025***	0.018***	0.027***	0.016***
	(0.004)	(0.004)	(0.004)	(0.004)
Age	0.006**	0.008**	0.012***	0.014***
	(0.003)	(0.003)	(0.003)	(0.004)
Size	−0.002	0.001	−0.009***	−0.007***
	(0.002)	(0.002)	(0.002)	(0.002)
Leverage	−0.026**	−0.019	0.002	0.012
	(0.011)	(0.012)	(0.011)	(0.012)
Share	−0.000	−0.000	−0.000	0.000
	(0.000)	(0.000)	(0.000)	(0.000)
TFP	0.004	−0.002	0.009***	0.002
	(0.003)	(0.003)	(0.003)	(0.004)
Digital	0.000***	0.001	0.000***	0.001
	(0.000)	(0.001)	(0.000)	(0.002)
Number of clusters (City)	252			
Observations	10,593	10,556	10,593	10,556
R-squared	0.009	0.064	0.012	0.060
Firm FE	No	Yes	No	Yes
Year FE	No	Yes	No	Yes
Prov×Year FE	No	Yes	No	Yes
Ind × Year FE	No	Yes	No	Yes

***, **, and * denote statistical significance at the 1, 5, and 10% levels, respectively.

#### 4.3.4 Wild cluster bootstrap estimation

This section examines whether the main findings remain robust to model specification choices and potential biases in standard error estimation. Two complementary robustness checks are employed: the Wild Cluster Bootstrap procedure and the Driscoll–Kraay heteroskedasticity and autocorrelation consistent (HAC) standard errors. The first addresses inference reliability when the number of clusters is limited or when intra-cluster correlation is pronounced, while the second accounts for cross-sectional dependence and serial correlation in panel data.

Following Cameron et al. (54), the Wild Cluster Bootstrap-t method resamples residuals within clusters to generate empirical *p*-values and confidence intervals that are robust to small sample cluster bias. A total of 9,999 bootstrap replications are conducted at the city level. The estimation results, reported in Table 8, show that the DID coefficients remain significantly positive for both Green R&D Efficiency (GrdE) and Green Transformation Efficiency (GopE). The corresponding 95% confidence intervals are [0.003986, 0.02299] and [0.004873, 0.02774], indicating that the policy continues to exert a substantial positive impact on firms’ green innovation efficiency even after accounting for small-sample clustered inference bias.

**Table 8:** Wild cluster bootstrap results (clustered by city, 9,999 replications).

Variable	DID coefficient	Std. error	*p*-value	95% confidence interval (lower, upper)
*GrdE*	0.013	0.005	0.004	[0.003986, 0.02299]
*GopE*	0.017	0.006	0.009	[0.004873, 0.02774]

The table reports Wild Cluster Bootstrap-t estimates based on 9,999 replications. Standard errors are clustered at the city level. All regressions control for firm size, firm age, ownership concentration, total factor productivity (TFP), digitalization intensity, and year fixed effects.

**Figure 10:**
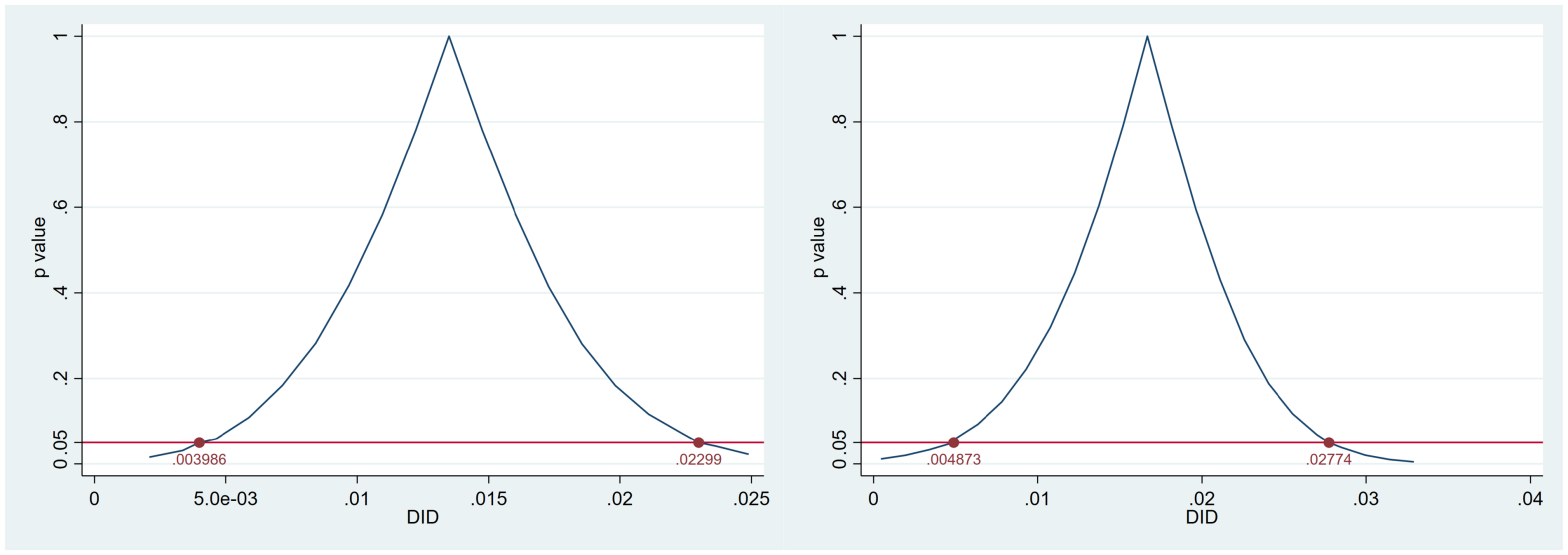
Wild cluster bootstrap estimates of DID effects on green innovation efficiency.

The bootstrap distributions illustrated in Figure 10 provide additional visual evidence of robustness. The horizontal axis represents the estimated DID coefficients, and the vertical axis displays the kernel density of bootstrap replications. The vertical dashed line denotes the zero benchmark, while the shaded region represents the 95% confidence interval. For both efficiency measures, the distributions are concentrated to the right of zero, reinforcing the robustness and statistical significance of the estimated treatment effects. To further account for cross-sectional dependence and serial correlation in panel data, we complement this analysis with Driscoll–Kraay robust standard errors.

#### 4.3.5 Driscoll–Kraay robust standard errors

An additional robustness assessment re-estimates the baseline model using Driscoll–Kraay heteroskedasticity and autocorrelation consistent (HAC) standard errors, which account for potential cross-sectional dependence and serial correlation (55). This estimator provides consistent inference in panels with both temporal and spatial correlations, making it particularly suitable for multi-period regional policy evaluations. Table 9 presents the estimation results. The coefficients on the DID variable remain positive and statistically significant at the 1% level for both Green R&D Efficiency (GrdE) and Green Output Efficiency (GopE). The estimated magnitudes are broadly comparable to the baseline findings, indicating that the policy exerts a robust positive influence on both the generation and transformation dimensions of green innovation. These outcomes reinforce the reliability of the baseline inference and demonstrate that the estimated effects are not driven by specific assumptions about error structure or inference procedures. Jointly, the results from the Wild Cluster Bootstrap and Driscoll–Kraay estimations provide consistent evidence that the DID estimates are stable across alternative inference procedures. The policy intervention significantly enhances firm-level green innovation efficiency, and this conclusion holds even under more flexible error structures that account for small-sample clustering bias, autocorrelation, and cross-sectional dependence. Having verified inference robustness under various statistical frameworks, we next validate the results by adopting alternative measures of green innovation to confirm that the positive effects of the AIDP are not model-dependent.

**Table 9:** Driscoll–Kraay robust standard errors.

Variables	(1)	(2)
	*GrdE*	*GopE*
DID	0.014***	0.017***
	(0.003)	(0.002)
Age	0.012*	0.015**
	(0.006)	(0.005)
Size	−0.005*	−0.007***
	(0.003)	(0.002)
Leverage	−0.015**	−0.012
	(0.006)	(0.009)
Share	−0.000	0.000
	(0.000)	(0.000)
TFP	0.008***	0.008***
	(0.001)	(0.002)
Digital	0.000*	0.000
	(0.000)	(0.000)
Constant	0.014***	0.017***
	(0.003)	(0.002)
Clusters	Driscollb Kraay SE	Driscollb Kraay SE
Observations	23,682	23,682
R-squared	0.359	0.361
Number of groups	3,094	3,094

Driscoll–Kraay HAC standard errors are computed with two lags. All specifications include firm-level controls and year fixed effects. The results are robust to cross-sectional dependence and serial correlation.

#### 4.3.6 Alternative outcome measures

Another robustness check is conducted using alternative measures of green innovation. While the baseline outcomes—green R&D efficiency (GrdE) and green innovation output efficiency (GopE)—capture firm-level performance through a multi-stage production process, they rely on efficiency frontier estimation, which may be sensitive to functional form assumptions or measurement errors in intermediate inputs. Consistency of the results is further assessed by replacing the baseline outcomes with two widely used alternative indicators.

The first proxy for green innovation, LnGreenPatent , is measured as the logarithm of firm-level green patent applications within a year, where green patents are identified according to WIPO’s environmental classification scheme. This provides an objective measure of innovation output. As a complement, we construct a dynamic indicator, LnGreenInnovation, which captures both the intensity and persistence of innovation activities. Specifically, it is computed by multiplying the growth rate of green patent filings across consecutive periods (t − 2 to t − 1 and t − 1 to t) with the firm’s patent stock in the corresponding period, thereby integrating both scale and continuity into the measurement of green innovation.

Table 10 reports the results. The estimated coefficients of the DID variable remain positive and statistically significant across all model specifications. Specifically, the implementation of the AI Pilot Zone policy increases LnGreenPatent by 0.148–0.478 and LnGreenInnovation by 0.154–0.365. These results hold after including firm and year fixed effects and a comprehensive set of control variables. Overall, the findings indicate that the policy based on AI consistently promotes green innovation, regardless of the measure used, reinforcing the reliability and robustness of the core conclusion. Full details of standard errors, city-level clustering, and robustness to outliers are provided to enhance transparency. Verifying the results with alternative dependent variables confirms that the observed positive effects of the AI Pilot Zones reflect a genuine policy induced improvement in firms’ green innovation performance, rather than artifacts of model specification or particular variable constructions. To further eliminate confounding influences, we finally control for concurrent environmental and innovation oriented city-level policies that might overlap with the AI Pilot Zone initiative.

**Table 10:** Supplementary Outcome Analysis: Green Patent Output.

Variables	(1)	(2)	(3)	(4)
	*LnGreenPatent*	*LnGreenPatent*	*LnGreenInnovation*	*LnGreenInnovation*
DID	0.478***	0.148***	0.365***	0.154**
	(0.027)	(0.048)	(0.037)	(0.074)
Age	−0.125***	−0.139***	−0.192***	−0.428***
	(0.013)	(0.036)	(0.021)	(0.076)
Size	0.497***	0.494***	0.558***	0.661***
	(0.011)	(0.050)	(0.015)	(0.064)
Leverage	0.084	−0.229*	0.117	−0.480**
	(0.063)	(0.127)	(0.087)	(0.199)
Share	−0.005***	0.000	−0.006***	−0.003
	(0.001)	(0.002)	(0.001)	(0.003)
TFP	0.066***	0.016	0.108***	−0.010
	(0.019)	(0.038)	(0.026)	(0.063)
Digital	0.000***	−0.011**	0.000*	−0.005
	(0.000)	(0.004)	(0.000)	(0.008)
Number of clusters (City)	260			
Observations	23,682	23,682	23,682	23,682
R-squared	0.305	0.764	0.220	0.634
Firm FE	Yes	Yes	Yes	Yes
Year FE	Yes	Yes	Yes	Yes
Prov × Year FE	Yes	Yes	Yes	Yes
Ind × Year FE	Yes	Yes	Yes	Yes

This table presents robustness checks employing alternative proxies for green innovation. In Columns (1–2), the dependent variable is the natural logarithm of the total number of green patent applications (LnGreenPatent). Columns (3–4) instead adopt LnGreenInnovation, defined as the year-on-year growth rate of total green patent applications between consecutive periods (t–2, t–1) and (t–1, t), weighted by the corresponding patent counts in t–1 and t. This measure captures both the intensity and the persistence of firms’ green innovation activities. Statistical significance is denoted at the 1, 5, and 10% levels by ***, **, and *, respectively.

#### 4.3.7 Controlling for concurrent city-level policies

An additional robustness check incorporates the Low Carbon City Pilot (LCCP) program as a control variable to account for potential bias from overlapping cities policies. In 2010, China’s National Development and Reform Commission (NDRC) launched the LCCP initiative to promote low-carbon industrial structures, improve energy efficiency, and support green urban development. Three rounds of pilot cities were announced in 2010, 2017, and 2023. Because the Low Carbon City Pilot shares objectives similar to the Artificial Intelligence Development Pilot (AIDP), including promoting green innovation and sustainable industrial transformation, its implementation may coincide with AIDP exposure and confound the estimated treatment effects. All listed firms located in pilot cities or provinces are coded as LCCP = 1, while others are coded as 0, and this variable is included in the regression specification.

Table 11 reports the results after controlling for the Low Carbon City Pilot. The estimated coefficients of the DID variable remain positive and statistically significant at the 1 and 5% levels for both green R&D efficiency (GrdE) and green innovation output efficiency (GopE). Specifically, the estimated effect of AIDP on green R&D efficiency is 0.018 (*p* < 0.01), and the effect on green output efficiency is 0.014 (*p* < 0.05). These results indicate that the AIDP continues to significantly enhance both green technology input and output efficiency, even after accounting for concurrent cities low carbon policy interventions. The supplementary analyses further confirm that the main findings are not driven by overlapping environmental or innovation-focused policy pilots. Overall, the positive impact of the AI Pilot Zones on corporate green innovation performance remains robust, reinforcing the validity of the identification strategy and the reliability of the estimated causal effects.

**Table 11:** Robustness check controlling for the low-carbon city pilot policy.

Variables	(1)	(2)
	*GrdE*	*GopE*
DID	0.018***	0.014**
	(0.006)	(0.006)
Age	0.009*	0.013***
	(0.006)	(0.005)
Size	−0.005	−0.008**
	(0.003)	(0.004)
Leverage	−0.016	−0.014
	(0.013)	(0.011)
Share	−0.000	−0.000
	(0.000)	(0.000)
TFP	0.008**	0.009**
	(0.004)	(0.004)
Digital	0.001	0.001
	(0.001)	(0.001)
LCCP	0.003	−0.001
	(0.004)	(0.004)
Number of clusters (City)	260	
Observations	23,682	23,682
R-squared	0.561	0.557
Firm FE	Yes	Yes
Year FE	Yes	Yes
Prov×Year FE	Yes	Yes
Ind × Year FE	Yes	Yes

Robust standard errors in parentheses. ***p < 0.01, **p < 0.05, *p < 0.1.

## 5 Mechanism analysis

This section examines the internal mechanisms through which the AIDP policy affects green innovation efficiency, focusing on financial, technological, and governance factors. The analysis examines how the Artificial Intelligence Innovation and Development Pilot Zones (AIDP) influence firm’ green innovation efficiency (GIE) within a sequential mediation framework. The policy is hypothesized to affect firm’ level mechanisms that subsequently shape green innovation outcomes. Conceptually, the causal pathway progresses from policy implementation to firm’ specific channels, including financial conditions, technological capacity, and governance quality, through which the AIDP impacts GIE. This approach emphasizes the mediating mechanisms rather than conditional heterogeneity, clarifying the internal process linking the policy to innovation performance. To begin the mechanism analysis, we first explore the direct impact of AIDP on intermediate firm-level variables that reflect financial, technological, and governance conditions.

In the first stage of the mechanism, the effects of AIDP on key intermediate variables reflecting financial constraints, technological upgrading, and governance improvements are examined. The results, presented in Table 12, indicate that firms located in pilot zones face lower financial frictions, as reflected in a decline in the Whited Wu index. This finding suggests that the policy improves access to credit and alleviates external financing limitations, enabling firms to undertake long-term and higher-risk green research and development projects. In addition, AIDP significantly increases firms’ investments related to AI, operating revenue, and current assets, showing an enhancement of internal resources and digital capabilities. These improvements allow firms to finance innovation from their own resources, accelerate technology adoption, and increase absorptive capacity for advanced green technologies. Furthermore, AIDP is associated with lower operating costs and higher environmental, social, and governance performance, reflecting better operational efficiency and management quality. Overall, the pilot zones contribute not only to a more favorable external financing environment but also to improved internal management and sustainability practices.

**Table 12:** Mechanism analysis of AIDP’s impact on green innovation efficiency.

Variables	(1)	(2)	(3)	(4)	(5)	(6)	(7)
	*WW*	*Revenue*	*AISoftInvest*	*AIHardInvest*	*CurrAssets*	*Cost*	*ESG*
DID	0.025**	0.006	0.152***	0.278***	0.124**	0.006	0.160***
	(0.011)	(0.004)	(0.055)	(0.088)	(0.048)	(0.004)	(0.031)
Number of clusters (City)			260				
Observations	23,682	23,682	18,667	9,676	23,682	23,682	23,682
R-squared	0.226	0.947	0.707	0.776	0.801	0.950	0.594
Firm FE	Yes	Yes	Yes	Yes	Yes	Yes	Yes
Year FE	Yes	Yes	Yes	Yes	Yes	Yes	Yes
Prov × Year FE	Yes	Yes	Yes	Yes	Yes	Yes	Yes
Ind × Year FE	Yes	Yes	Yes	Yes	Yes	Yes	Yes

***, **, and * denote statistical significance at the 1, 5, and 10% levels, respectively.

The second stage links these mechanism variables to innovation efficiency at individual firms to identify their mediating roles. The results in Table 13 indicate that AI related investment, liquidity, and environmental, social, and governance performance are all positively and significantly associated with green innovation efficiency. When these mechanism variables are included in the analysis, the estimated effect of AIDP on innovation efficiency is reduced, which is consistent with partial mediation. This pattern suggests that the policy enhances green innovation efficiency not only by directly encouraging innovation but also by transforming financial, technological, and governance conditions that support sustainable innovation.

**Table 13:** Mechanism mediation of AIDP on green innovation efficiency.

Variables	(1)	(2)	(3)	(4)	(5)	(6)	(7)	(8)	(9)	(10)	(11)	(12)	(13)	(14)
	*GrdE*	*GrdE*	*GrdE*	*GrdE*	*GrdE*	*GrdE*	*GrdE*	*GopE*	*GopE*	*GopE*	*GopE*	*GopE*	*GopE*	*GopE*
DID	0.061***	0.054***	0.050***	0.053***	0.049***	0.054***	0.053***	0.060***	0.053***	0.050***	0.051***	0.049***	0.053***	0.053***
	(0.003)	(0.003)	(0.003)	(0.005)	(0.004)	(0.003)	(0.003)	(0.003)	(0.003)	(0.003)	(0.005)	(0.004)	(0.003)	(0.003)
WW	−0.000							0.003						
	(0.002)							(0.002)						
Revenue		0.001							0.008					
		(0.012)							(0.012)					
AISoftInvest			0.001							0.002				
			(0.002)							(0.002)				
AIHardInvest				0.001*							0.001			
				(0.001)							(0.001)			
CurrAssets					0.000							0.002*		
					(0.001)							(0.001)		
Cost						0.000							0.008	
						(0.013)							(0.013)	
ESG							0.003**							0.002*
							(0.001)							(0.001)
Constant	0.258***	0.281***	0.309***	0.252***	0.280***	0.281***	0.284***	0.314***	0.346***	0.365***	0.363***	0.378***	0.346***	0.344***
	(0.021)	(0.021)	(0.025)	(0.036)	(0.034)	(0.021)	(0.020)	(0.021)	(0.021)	(0.025)	(0.036)	(0.034)	(0.021)	(0.020)
Observations	23,682	23,682	18,667	18,667	23,682	23,682	23,682	23,682	23,682	23,682	23,682	23,682	23,682	23,682
R-squared	0.369	0.359	0.353	0.353	0.346	0.359	0.359	0.375	0.361	0.357	0.356	0.342	0.361	0.361
Controls	Yes	Yes	Yes	Yes	Yes	Yes	Yes	Yes	Yes	Yes	Yes	Yes	Yes	Yes
Firm FE	Yes	Yes	Yes	Yes	Yes	Yes	Yes	Yes	Yes	Yes	Yes	Yes	Yes	Yes
Year FE	Yes	Yes	Yes	Yes	Yes	Yes	Yes	Yes	Yes	Yes	Yes	Yes	Yes	Yes
Prov×Year FE	Yes	Yes	Yes	Yes	Yes	Yes	Yes	Yes	Yes	Yes	Yes	Yes	Yes	Yes
Ind × Year FE	Yes	Yes	Yes	Yes	Yes	Yes	Yes	Yes	Yes	Yes	Yes	Yes	Yes	Yes

Robust standard errors clustered at city level are reported in parentheses. ***p < 0.01, **p < 0.05, *p < 0.1.

The findings illustrate a coherent process: AIDP first relaxes financial constraints and encourages investment in digital technologies, which improves liquidity, reduces operating costs, and enhances governance performance. These improvements strengthen firms’ capacity for green innovation and lead to better environmental outcomes. The three mechanisms reinforce each other: improved digital capabilities increase operational efficiency and governance quality, which further reduces financing costs and facilitates additional investment in green innovation. Together, these results support a mediation-based interpretation, demonstrating that AIDP promotes green innovation efficiency through interconnected financial, technological, and governance channels.

## 6 Discussion

This section discusses the heterogeneous effects of the AIDP policy across ownership types, sectors, and factor intensities, providing insights for differentiated policy design. Building on the mechanism analysis, this section investigates whether the effects of AIDP differ across firm ownership types, industrial sectors, and factor intensities. We extend the analysis by examining heterogeneous effects of the Artificial Intelligence Innovation and Development Pilot Zone (AIDP) policy on firms’ green innovation efficiency (GIE) across different subsamples. Investigating these heterogeneous responses is crucial because firms differ in their institutional environments, resource endowments, and operational characteristics, all of which affect their capacity to implement artificial intelligence technologies effectively. The impact of the policy also varies by ownership type, industrial sector, and technological or input intensity, reflecting differences in managerial flexibility, innovation incentives, and readiness for digital transformation. We pre-defined these three dimensions of heterogeneity to capture the most relevant sources of variation in firms’ responsiveness. Understanding these heterogeneous effects not only clarifies the scope and mechanisms of AIDP’s influence but also provides nuanced insights for policymakers aiming to target differentiated groups of firms while evaluating broader societal benefits, including public health outcomes. Bonferroni and false discovery rate (FDR) adjustments are applied to account for multiple hypothesis testing across subgroups, and results that remain significant after these corrections are marked with † in the Appendix.

We first examine ownership heterogeneity to assess whether state-owned and non-state-owned enterprises respond differently to the AIDP. Heterogeneity (Table 14) by ownership type reveals that privately owned and foreign-invested firms demonstrate the strongest and most consistent positive responses in both green research and development efficiency and green innovation output efficiency. Local state-owned enterprises (SOEs) and central SOEs exhibit weaker or insignificant effects. The difference persists after accounting for multiple testing corrections. Specifically, the estimated effect on green research and development efficiency for non-state-owned firms is statistically significant at 0.027 (†), whereas local and central SOEs show no significant changes. For green innovation output efficiency, non-state-owned firms and central SOEs experience significant positive effects, while local SOEs show negligible or even negative effects. These patterns suggest that non-state-owned firms are more capable of leveraging policies associated with artificial intelligence, likely due to stronger innovation incentives, greater managerial autonomy, and heightened responsiveness to market signals. From a public health perspective, these firms’ responsiveness indicates a higher potential for adopting cleaner production methods, thereby reducing pollution-related health risks, such as respiratory illnesses and chronic conditions.

**Table 14:** Heterogeneity analysis by ownership type.

Variables	(1)	(2)	(3)	(4)	(5)	(6)
	*GrdE*	*GrdE*	*GrdE*	*GopE*	*GopE*	*GopE*
	Local SOEs	Central SOEs	Non-SOEs	Local SOEs	Central SOEs	Non-SOEs
DID	0.020	−0.010	0.027***†	−0.019	0.096***†	0.019***†
	(0.013)	(0.085)	(0.008)	(0.012)	(0.035)	(0.007)
Observations	7,091	885	14,197	7,091	885	14,197
R-squared	0.601	0.704	0.562	0.614	0.715	0.548
Firm FE	Yes	Yes	Yes	Yes	Yes	Yes
Year FE	Yes	Yes	Yes	Yes	Yes	Yes
Prov × Year FE	Yes	Yes	Yes	Yes	Yes	Yes
Ind × Year FE	Yes	Yes	Yes	Yes	Yes	Yes

This table reports heterogeneity results of the AI Pilot Zone Policy by firm ownership. Enterprises are divided into three categories: (i) Local SOEs, referring to state-owned firms under provincial or municipal control; (ii) Central SOEs, which are directly administered by the central government; and (iii) Non-SOEs, encompassing privately owned and foreign-invested companies. ***, **, and * indicate statistical significance at the 1, 5, and 10% levels, respectively. The symbol † indicates results that remain significant after Bonferroni and false discovery rate (FDR) adjustments for multiple hypothesis testing across subgroups.

After identifying ownership-based differences, we next explore whether these patterns vary across industries with different technological characteristics and emission profiles. Industrial heterogeneity analysis (Table 15) further confirms that the AIDP policy has the most pronounced impact in the manufacturing sector, consistent with expectations since these firms are early adopters of automation and digital technologies. Significant positive effects also appear in construction and business services sectors, albeit with varying magnitudes. In contrast, sectors such as mining and utilities exhibit negligible or negative effects. These findings suggest that improvements in green innovation are more likely in industries characterized by scalable production, high potential for digital adoption, and complex supply chains capable of benefiting from artificial intelligence optimization. Given that manufacturing and construction are major contributors to industrial emissions, the positive gains in these sectors imply substantial public health benefits, including improved urban air quality and reduced exposure to harmful particulates in densely populated areas.

**Table 15:** Heterogeneity analysis by across industry categories.

Variables	(1)	(2)	(3)	(4)	(5)	(6)	(7)	(8)	(9)	(10)	(11)	(12)
	*GrdE*	*GrdE*	*GrdE*	*GrdE*	*GrdE*	*GrdE*	*GopE*	*GopE*	*GopE*	*GopE*	*GopE*	*GopE*
	B	C	D	E	G	K	B	C	D	E	G	K
DID	−0.041	0.019***†	0.011	0.033	−0.107***†	0.007	−0.123	0.014**†	0.006	−0.018	0.054	0.013
	(0.046)	(0.007)	(0.053)	(0.099)	(0.039)	(0.034)	(0.107)	(0.007)	(0.030)	(0.082)	(0.061)	(0.027)
Observations	549	19,259	787	632	684	836	549	19,259	787	632	684	836
R-squared	0.601	0.704	0.562	0.614	0.715	0.548	0.601	0.704	0.562	0.614	0.715	0.548
Firm FE	Yes	Yes	Yes	Yes	Yes	Yes	Yes	Yes	Yes	Yes	Yes	Yes
Year FE	Yes	Yes	Yes	Yes	Yes	Yes	Yes	Yes	Yes	Yes	Yes	Yes
Prov × Year FE	Yes	Yes	Yes	Yes	Yes	Yes	Yes	Yes	Yes	Yes	Yes	Yes
Ind × Year FE	Yes	Yes	Yes	Yes	Yes	Yes	Yes	Yes	Yes	Yes	Yes	Yes

This table reports the heterogeneous effects of the AI Pilot Zone Policy across industries. Industry classification follows the national standard codes: B = Mining; C = Manufacturing; D = Electricity, Heat, Gas, and Water Supply; E = Construction; G = Wholesale and Retail Trade; and K = Leasing and Business Services. Statistical significance is denoted by ***, **, and * at the 1, 5, and 10% levels, respectively. The symbol † indicates results that remain significant after Bonferroni and false discovery rate (FDR) adjustments for multiple hypothesis testing across subgroups.

Finally, heterogeneity across technological, capital, and labor intensity (Table 16) highlights that the policy positively influences most subgroups, though effect sizes vary. Both high-tech and low-tech firms experience improvements in green innovation efficiency, with slightly stronger gains in low-tech sectors, indicating that artificial intelligence facilitates overcoming innovation barriers in traditional industries. High and low capital intensive industries both benefit, with low capital intensive firms showing greater improvements in green research and development efficiency, suggesting that artificial intelligence can partially substitute for physical capital in innovation processes. Similarly, high and low labor intensive firms benefit, but the effect is more pronounced in high labor intensive firms, highlighting the role of artificial intelligence in upgrading labor intensive operations through automation and intelligent management systems. Because low-tech, labor intensive, and traditionally high emission industries often employ large workforces and have significant environmental impacts, improvements in green innovation efficiency within these groups generate extensive public health benefits, from cleaner industrial environments to reduced occupational hazards.

**Table 16:** Heterogeneity analysis by high-tech industry classification, capital intensity and labor intensity.

Variables	(1)	(2)	(3)	(4)	(5)	(6)	(7)	(8)	(9)	(10)	(11)	(12)
	*GrdE*	*GrdE*	*GrdE*	*GrdE*	*GrdE*	*GrdE*	*GopE*	*GopE*	*GopE*	*GopE*	*GopE*	*GopE*
	H-T	L-T	H-C	L-C	H-P	L-P	H-T	L-T	H-C	L-C	H-P	L-P
DID	0.017**†	0.019*†	0.001	0.020***†	0.027**†	0.012*†	0.016**†	0.019*†	0.003	0.018***†	0.025**†	0.015*
	(0.007)	(0.011)	(0.015)	(0.007)	(0.012)	(0.008)	(0.007)	(0.010)	(0.021)	(0.006)	(0.010)	(0.008)
Observations	15,234	8,076	4,997	18,291	6,793	16,432	15,234	8,076	4,997	18,291	6,793	16,432
R-squared	0.563	0.584	0.592	0.562	0.578	0.564	0.558	0.580	0.594	0.557	0.582	0.555
Firm FE	Yes	Yes	Yes	Yes	Yes	Yes	Yes	Yes	Yes	Yes	Yes	Yes
Year FE	Yes	Yes	Yes	Yes	Yes	Yes	Yes	Yes	Yes	Yes	Yes	Yes
Prov × Year FE	Yes	Yes	Yes	Yes	Yes	Yes	Yes	Yes	Yes	Yes	Yes	Yes
Ind × Year FE	Yes	Yes	Yes	Yes	Yes	Yes	Yes	Yes	Yes	Yes	Yes	Yes

This table presents heterogeneity analysis of the AI Pilot Zone Policy’s impact on green innovation across different types of industries. The sample is divided based on three dimensions: H-T / L-T: High-tech vs. Low-tech industries, based on national industry classifications. H-C / L-C: High capital-intensive vs. Low capital-intensive industries, de-fined by the top and bottom quantiles of capital–labor ratios. H-P / L-P: High labor-intensive vs. Low labor-intensive industries, classified by employment-to-output ratios. ***, **, and * indicate statistical significance at the 1, 5, and 10% levels, respectively. The symbol † indicates results that remain significant after Bonferroni and false discovery rate (FDR) adjustments for multiple hypothesis testing across subgroups.

Overall, the heterogeneity analysis confirms that the policy’s effects are unevenly distributed across firms and industries, revealing differentiated pathways to environmental and health benefits. Firms and sectors that respond more strongly to policies based on artificial intelligence are better positioned to translate innovation gains into cleaner production, reduced emissions, and improved community well-being. Robustness checks using Bonferroni and FDR corrections indicate that positive effects, particularly for non-state-owned enterprises, manufacturing, low-capital-intensive, and high labor-intensive firms, are unlikely to be false discoveries. These findings underscore the dual benefits of the policy: targeted digital transformation accelerates sustainable innovation while simultaneously supporting public health through pollution reduction and enhanced environmental quality.

## 7 Conclusion

This section summarizes the key findings, highlights their policy implications, and suggests directions for future research on green innovation and public health outcomes. This study provides strong empirical evidence that the Artificial Intelligence Innovation and Development Pilot Zones (AIDP) have effectively enhanced green innovation efficiency among Chinese firms. Using a rigorous difference-in-differences approach applied to panel data from 2011 to 2022, we find that firms located in cities designated as AIDP significantly improve both their green research and development efficiency and green innovation output compared with firms outside these zones. These effects remain significant across multiple model specifications and placebo tests, indicating that the AIDP policy itself is the primary driver of these improvements. This finding is consistent with theoretical perspectives suggesting that targeted innovation zones create environments that reduce barriers and foster resource synergies necessary for sustainable technological advancement. Beyond economic and technological benefits, the improvement in green innovation efficiency contributes to broader societal gains. By reducing industrial emissions and promoting cleaner production processes, the policy helps mitigate environmental pollution, which generates substantial public health benefits through lower exposure to pollution related risks. It should be noted, however, that these public health benefits are conceptually inferred rather than empirically measured, as the available data do not include direct health outcomes. Future research could strengthen this dimension by incorporating proxy health indicators, such as pollution related mortality, morbidity, or healthcare expenditure, to empirically validate the connection between environmental improvements and public health outcomes.

The mechanisms underlying these improvements reveal a multi-dimensional transformation catalyzed by the AIDP policy. Mechanism tests indicate that the policy enhances green innovation efficiency primarily through capital-intensive investment in artificial intelligence hardware and improvements in firms’ liquidity positions. Both channels strengthen firms’ capacity to sustain long-term and uncertain green innovation activities. By fostering greater liquidity and easing operational cash flow constraints, the policy indirectly enhances firms’ absorptive capacity and technological adaptability. Furthermore, the policy significantly improves corporate environmental, social, and governance performance, representing a governance and sustainability channel through which the AIDP supports greener corporate transformation and accountability. Among these channels, investment in artificial intelligence hardware acts as the pivotal driver linking digital transformation to green innovation. By improving production efficiency, enabling real-time environmental monitoring, and strengthening transparency in governance and sustainability practices, hardware-based investment amplifies the positive effects of liquidity and governance improvements, generating synergistic gains for green innovation outcomes. Together, these governance and efficiency improvements enhance firms’ competitiveness while contributing to more sustainable and resilient industrial structures. Overall, empirical evidence underscores that the improvement of green innovation efficiency under the AIDP policy is primarily rooted in technological upgrading, financial flexibility, and sustainability governance, rather than cost reductions or expansion of soft investments.

The heterogeneous effects identified also imply differentiated public health benefits. Sectors such as manufacturing and labor-intensive industries, where policy impacts are strongest, are simultaneously those with historically higher emissions and occupational health risks. Targeting these sectors not only maximizes gains in green innovation but also produces disproportionately large reductions in pollution exposure, respiratory illnesses, and work-related health hazards. In contrast, weaker responses in mining and utility sectors suggest that additional, sector-specific interventions are required to achieve comparable health improvements. Tailoring policy design to these differentiated health benefits can help optimize resource allocation, ensuring that digital transformation policies deliver maximum joint benefits for environmental sustainability and population health. Equitable policy expansion should ensure access to resources and capacity-building support for less developed regions to prevent widening regional disparities in innovation capacity and health outcomes.

Importantly, the heterogeneous effects we identify also imply differentiated public health benefits. Sectors such as manufacturing and labor-intensive industries, where policy impacts are strongest, are simultaneously those with historically higher pollution emissions and occupational health risks. Targeting these sectors not only maximizes gains in green innovation but also produces disproportionately large reductions in pollution exposure, respiratory illnesses, and work-related health hazards. By contrast, the weaker responses observed in mining and utilities suggest that additional, sector-specific interventions are needed to secure comparable health improvements. Tailoring policy design to these differentiated health benefits can therefore help optimize resource allocation, ensuring that digital transformation policies deliver the greatest possible joint dividends for both environmental sustainability and population health. To prevent widening regional disparities in innovation capacity and health benefits, policy expansion must ensure equitable access to resources and capacity-building support for less-developed regions.

Policy implications emerge from these findings. Significant mediating effects mainly arise from investment in artificial intelligence hardware, liquidity improvements, and enhancements in environmental, social, and governance practices. Policy design should therefore prioritize capital-intensive digital infrastructure projects and governance upgrading as central levers for driving green innovation. Support for software investments or operational cost reductions alone may not yield comparable outcomes. Policymakers should integrate hardware investment with green finance instruments, including low-interest loans, sustainability linked bonds, and targeted subsidies for environmentally oriented technologies, to reduce financial barriers and accelerate clean technology adoption. Ensuring liquidity support in less developed regions can prevent regional disparities in innovation. Furthermore, the sequencing of artificial intelligence investment and governance policies should be regionally differentiated, with infrastructure and liquidity support prioritized in less developed regions before introducing complex governance frameworks, promoting inclusive green transformation. Clear governance-based performance metrics within artificial intelligence policy frameworks can safeguard against environmentally detrimental growth and ensure that technological upgrading aligns with decarbonization and sustainable production objectives.

Despite the robustness of these findings, this study acknowledges several limitations. Although the difference-in-differences methodology controls for time-invariant unobserved factors, it may not fully account for dynamic confounders or overlapping policies affecting innovative outcomes. More refined identification strategies, such as triple-difference methods or matched sampling, could strengthen causal interpretations. Reliance on patent-based indicators of green innovation, though widely accepted, may underestimate the full environmental and market impact of new technologies. Incorporating firm’ environmental performance metrics, product innovation data, and supply chain effects would provide a more holistic assessment of policy outcomes. Future studies could also empirically test the conceptual link between environmental improvements and public health by introducing direct or proxy health indicators, allowing a comprehensive evaluation of societal benefits from policies based on artificial intelligence. Lastly, because this analysis focuses exclusively on China, comparative studies across countries with different innovation systems and governance frameworks are necessary to evaluate the generalizability of artificial intelligence policies as drivers of sustainable innovation globally.

## Data Availability

The original contributions presented in the study are included in the article/Supplementary material, further inquiries can be directed to the corresponding author.
