# Supplementary material for: Artificial intelligence, green innovation efficiency, and public health benefits: evidence from China’s pilot zones with a DID approach
Source: Front Public Health. 2025 Nov 18;13:1713295. doi: 10.3389/fpubh.2025.1713295 (PMC12669213; doi:10.3389/fpubh.2025.1713295)
Supplement: Supplementary file 1 [file Table_1.docx]

**Appendix：**

**Appendix A. Supplementary Figures and Tables**


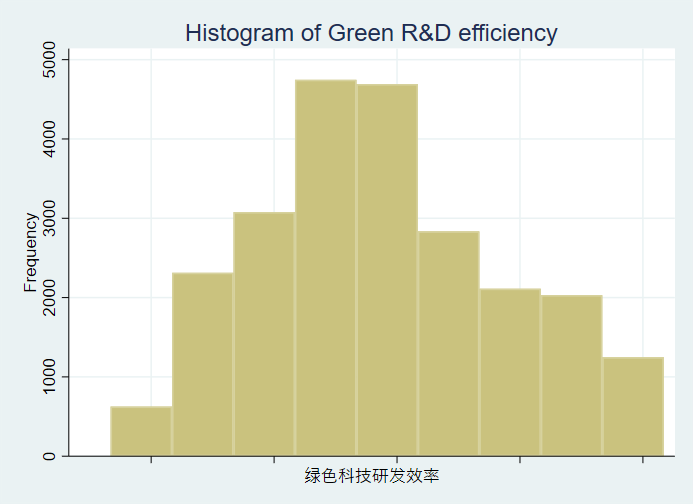

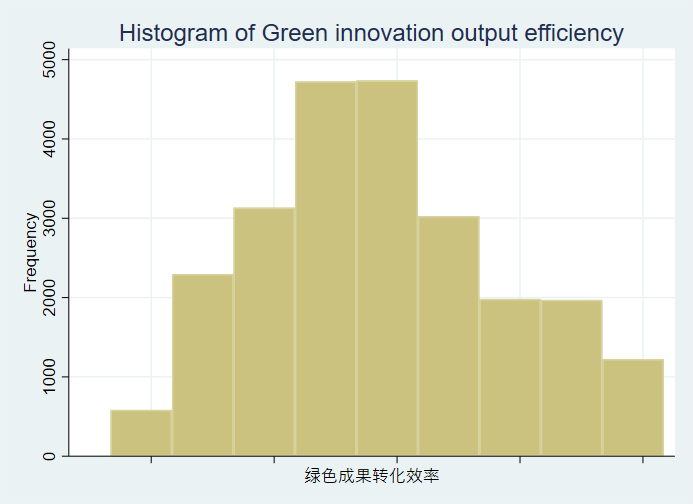


Supplement Figure 1. Histograms of Green R&D efficiency and Green innovation output efficiency


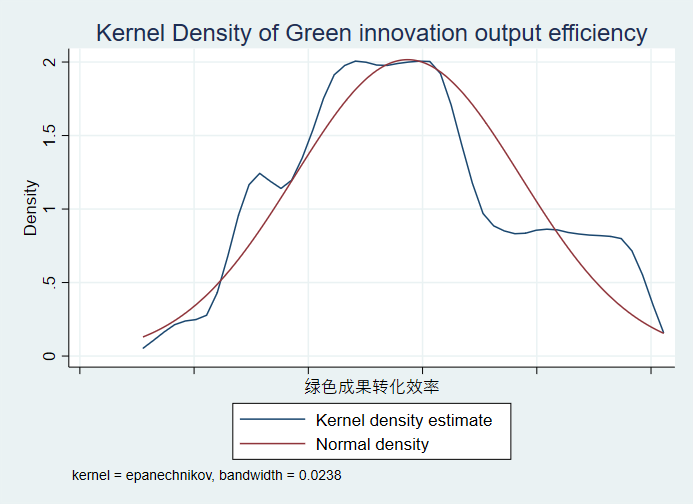

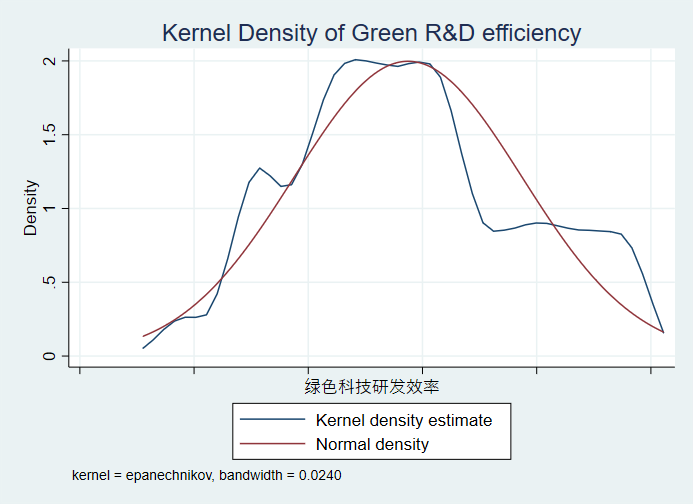


Supplement Figure 2. Kernel Density Plots

Supplement Table1. Matched Sample Composition Under Common Support

| Group | Number of Firms |
| --- | --- |
| Treated | 4,160 |
| Control | 19,514 |

**Appendix B. Measurement of Green Innovation Efficiency (GIE)**

Green innovation efficiency (GIE) constitutes the principal explained variable in this study, capturing firms’ capacity to generate and commercialize environmentally oriented technological knowledge under resource and environmental constraints.

B.1 Conceptual Framework

In line with the evolving literature on environmental productivity and innovation (Tone, 2001; Oh, 2010; Sueyoshi and Goto, 2019), GIE is decomposed into two interrelated components, Green R&D Efficiency and Green Innovation Output Efficiency, reflecting the two fundamental stages of the green innovation value chain: knowledge creation and commercial application.

The two-stage production structure allows the efficiency of the innovation process to be evaluated comprehensively, recognizing that technological development does not end with patent generation but extends to the diffusion and commercialization of green technologies. The first stage captures how effectively innovation inputs are transformed into intermediate technological outputs, while the second stage assesses the subsequent conversion of these intermediate outputs into final economic and environmental outcomes.

B.2 The Super-Efficiency SBM Model

Formally, the two-stage process is evaluated using the super-efficiency Slack-Based Measure (SBM) model, which corrects for slack in both inputs and outputs and allows the efficiency of fully efficient decision-making units (DMUs) to exceed unity. This approach enables a refined comparison of green innovation performance across firms and years.

To quantify GIE, we employ the super-efficiency Slack-Based Measure (SBM) model, which addresses the slackness issue inherent in traditional DEA models by incorporating slack variables into the objective function (58, 59), thus enhancing measurement precision. Importantly, the model accounts for undesirable outputs such as pollution, which is crucial for capturing the environmental dimension of innovation. The general form of the super-efficiency SBM model is as follows:

$$\min\left( \omega\right)^{*}=\frac{1+\frac{1}{\sigma}\sum_{i=1}^{\sigma} \frac{s_{i}^{-}}{A_{i0}^{t}}}{1-\frac{1}{\tau+\varphi}(\sum_{r=1}^{\tau} \frac{s_{r}^{+}}{B_{r0}^{t}}+\sum_{k=1}^{\varphi} \frac{s_{k}^{-}}{C_{k0}^{t}})} (1)$$

subject to:

$$s.t. \left\{ \begin{matrix} A_{i0}^{t}\geq\sum_{t=1}^{T} \sum_{j=1,\neq0}^{n} \mu_{j}^{t} A_{ij}^{t}-s_{i}^{-}i=1,2,\cdots,\sigma\\ B_{r0}^{t}\geq\sum_{t=1}^{T} \sum_{j=1,\neq0}^{n} \delta_{j}^{t} B_{rj}^{t}+s_{r}^{+}r=1,2,\cdots,\tau\\ \begin{matrix} C_{k0}^{t}\geq\sum_{t=1}^{T} \sum_{j=1,\neq0}^{n} \epsilon_{j}^{t} C_{kj}^{t}-s_{k}^{-}k=1,2,\cdots,\varphi\\ s_{i}^{-}\geq0(\forall i), {\mu_{j}^{t}\geq0(\forall j),s}_{r}^{+}\geq0(\forall r), s_{k}^{-}\geq0(\forall k), \end{matrix} \end{matrix} \right.$$

where $s_{i}^{-}$, $s_{r}^{+}$, and $s_{k}^{-}$are slack variables for inputs, desirable outputs, and undesirable outputs, respectively; $A$, $B$, and $C$denote observed values of inputs, desirable outputs, and undesirable outputs. This formulation allows us to comprehensively evaluate GIE while explicitly accounting for environmental impacts and regional heterogeneity.

**Appendix C. Green R&D Efficiency**

This appendix C evaluates firms’ ability to transform innovation related inputs into technological knowledge.

Inputs:

(1) Full time equivalent of R&D personnel;

(2) Internal R&D expenditure, deflated by the R&D price index and converted into capital stock.

Desirable Outputs:

(1) Total patent applications;

(2) Effective invention patents;

(3) Green patent applications (identified following the WIPO Green Inventory classification).

Undesirable Outputs:

Comprehensive environmental pollution index (constructed via entropy weighting based on five indicators: industrial SO₂ emissions, wastewater discharge, smoke and dust emissions, solid waste generation, and energy consumption per unit of industrial output).

**Appendix D. Green Innovation Output Efficiency**

This Appendix D quantifies how effectively intermediate technological outputs from Stage 1 are transformed into economic and environmental outcomes.

Inputs:

(1) Intermediate outputs from Stage 1 (patent applications and authorizations);

(2) Additional investments in new product development and technology assimilation (both deflated using the industrial producer price index).

Desirable Outputs:

(1) New product sales revenue;

(2) High-tech industrial output value.

Undesirable Outputs:

Comprehensive environmental pollution index (same construction as in Stage 1).

**Appendix E. Descriptive Visualization of Policy Effects**

This section presents a visual inspection of the policy’s association with the core outcome variables: Green R&D efficiency and Green innovation output efficiency.

To account for the time lag between innovation inputs and observable outcomes, a two-year lag structure is applied: inputs, intermediate outputs, and final outputs correspond to years $t$, $t+1$, and $t+2$, respectively. All monetary variables are deflated to constant 2015 prices, and indicators are normalized by firm size or total assets to ensure comparability across firms.

We begin our analysis with a visual inspection of the policy's association with the core outcome variables: Green R&D efficiency and Green innovation output efficiency. Supplement Figures 3 to 6 present bivariate scatterplots comparing the pre-policy mean (x-axis) against the post-policy mean (y-axis) for these efficiencies at both city and firm levels. The 45-degree line serves as a benchmark for no change; points lying above this line indicate an improvement in efficiency following the policy intervention.

A distinct spatial pattern emerges at the city level. Supplement Figure 3, which plots city-level Green R&D efficiency, reveals a dense cluster of points above the diagonal. This suggests a widespread, positive association between the policy and R&D performance across most cities, consistent with the policy's intended goal of stimulating green knowledge creation. In contrast, Supplement Figure 4, depicting city-level Green innovation output efficiency, shows a more dispersed distribution above the line. The greater variance indicates that the policy's link to the commercialization stage is more heterogeneous, potentially reflecting differing capacities among cities to translate R&D into marketable outputs.

This heterogeneity is markedly more pronounced at the micro level. Supplement Figure 5 for firm-level Green R&D efficiency displays significant scattering on both sides of the diagonal. While a majority of firms appear to have experienced gains, the substantial number of firms below the line underscores considerable firm-level divergence in R&D response, likely driven by differences in internal resources and absorptive capacity. The most striking heterogeneity is observed in Supplement Figure 6 for firm-level Green innovation output efficiency. The vast dispersion of points highlights the profound challenges firms face in the commercialization process, suggesting that the final step of transforming green patents into economic and environmental value is highly idiosyncratic and influenced by firm-specific constraints.

Collectively, these visualizations provide compelling descriptive evidence that motivates our subsequent causal identification strategy. They confirm that the policy period is associated with an upward shift in green innovation efficiency on average. More importantly, they underscore the critical importance of accounting for effect heterogeneity, across geographical units, organizational levels, and stages of the innovation pipeline, in any rigorous assessment of the policy's impact.


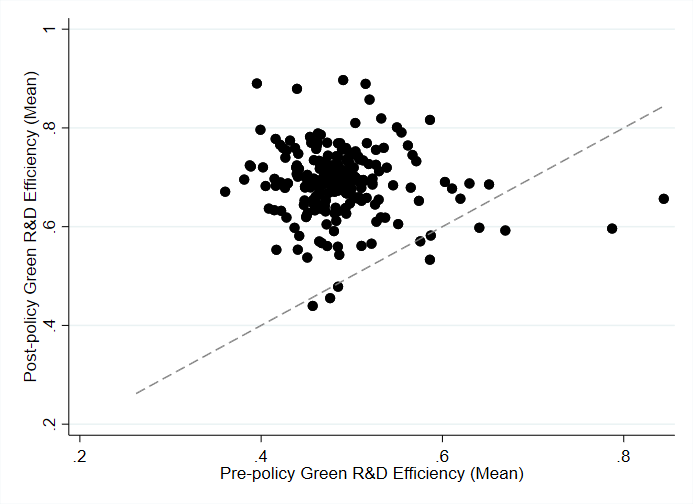


Supplement Figure 3. City-Level Green R&D Efficiency


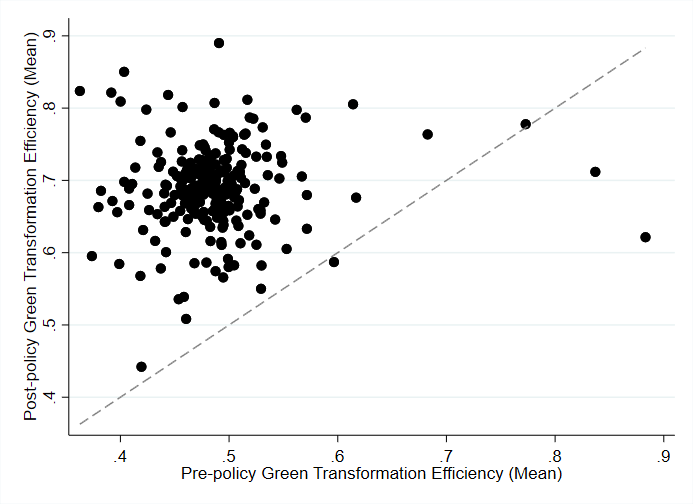


Supplement Figure 4. City-Level Green Innovation Output Efficiency


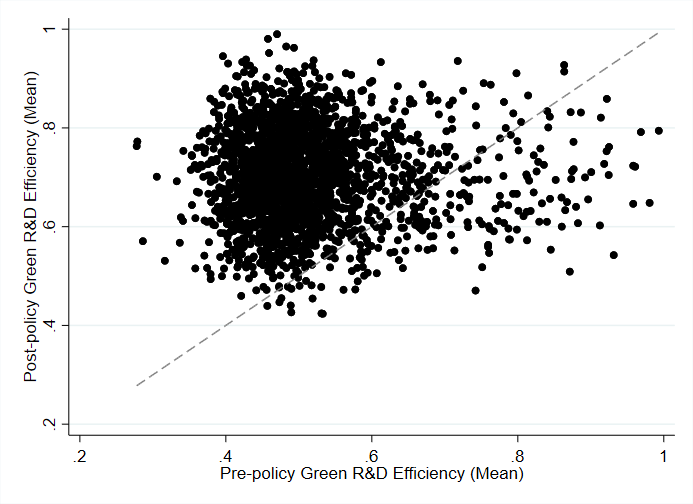


Supplement Figure 5. Firm-Level Green R&D Efficiency


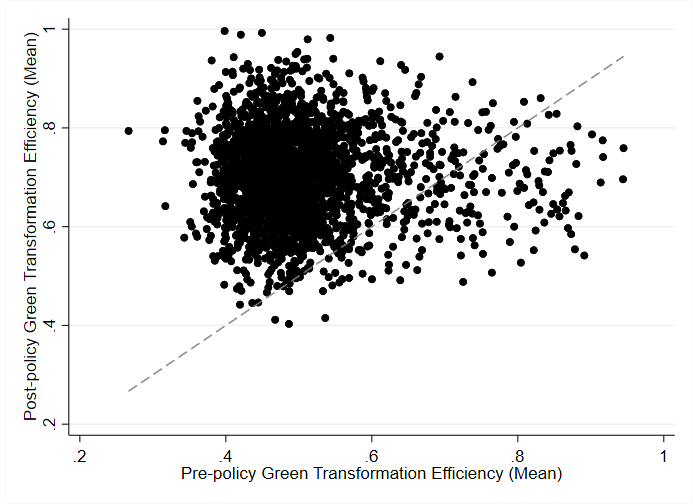


Supplement Figure 6. Firm-Level Green Innovation Output Efficiency

**Appendix F.** Bonferroni and FDR Adjusted

Supplement Table 2. DID Coefficients with Bonferroni and FDR Adjusted Significance

| **Table** | **Variable** | **DID** | **SE** | **p-value** | **Bonferroni p** | **FDR p** | **Bonferroni sig** | **FDR sig** |
| --- | --- | --- | --- | --- | --- | --- | --- | --- |
| 12 | Local SOEs GrdE | 0.020 | 0.013 | 0.120 | 0.720 | 0.120 | No | No |
| 12 | Central SOEs GrdE | -0.010 | 0.085 | 0.905 | 1.000 | 0.905 | No | No |
| 12 | Non-SOEs GrdE | 0.027 | 0.008 | 0.001 | 0.006 | 0.003 | Yes | Yes |
| 12 | Local SOEs GopE | -0.019 | 0.012 | 0.120 | 0.720 | 0.120 | No | No |
| 12 | Central SOEs GopE | 0.096 | 0.035 | 0.006 | 0.036 | 0.012 | Yes | Yes |
| 12 | Non-SOEs GopE | 0.019 | 0.007 | 0.005 | 0.030 | 0.010 | Yes | Yes |
| 13 | B GrdE | -0.041 | 0.046 | 0.374 | 1.000 | 0.374 | No | No |
| 13 | C GrdE | 0.019 | 0.007 | 0.006 | 0.072 | 0.012 | No | Yes |
| 13 | D GrdE | 0.011 | 0.053 | 0.837 | 1.000 | 0.837 | No | No |
| 13 | E GrdE | 0.033 | 0.099 | 0.740 | 1.000 | 0.740 | No | No |
| 13 | G GrdE | -0.107 | 0.039 | 0.006 | 0.072 | 0.012 | No | Yes |
| 13 | K GrdE | 0.007 | 0.034 | 0.837 | 1.000 | 0.837 | No | No |
| 13 | B GopE | -0.123 | 0.107 | 0.251 | 1.000 | 0.251 | No | No |
| 13 | C GopE | 0.014 | 0.007 | 0.042 | 0.504 | 0.042 | No | Yes |
| 13 | D GopE | 0.006 | 0.030 | 0.843 | 1.000 | 0.843 | No | No |
| 13 | E GopE | -0.018 | 0.082 | 0.829 | 1.000 | 0.829 | No | No |
| 13 | G GopE | 0.054 | 0.061 | 0.375 | 1.000 | 0.375 | No | No |
| 13 | K GopE | 0.013 | 0.027 | 0.638 | 1.000 | 0.638 | No | No |
| 14 | H-T GrdE | 0.017 | 0.007 | 0.016 | 0.192 | 0.016 | No | Yes |
| 14 | L-T GrdE | 0.019 | 0.011 | 0.085 | 1.000 | 0.085 | No | No |
| 14 | H-C GrdE | 0.001 | 0.015 | 0.946 | 1.000 | 0.946 | No | No |
| 14 | L-C GrdE | 0.020 | 0.007 | 0.004 | 0.048 | 0.004 | Yes | Yes |
| 14 | H-P GrdE | 0.027 | 0.012 | 0.024 | 0.288 | 0.024 | No | Yes |
| 14 | L-P GrdE | 0.012 | 0.008 | 0.129 | 1.000 | 0.129 | No | No |
| 14 | H-T GopE | 0.016 | 0.007 | 0.023 | 0.276 | 0.023 | No | Yes |
| 14 | L-T GopE | 0.019 | 0.010 | 0.060 | 1.000 | 0.060 | No | No |
| 14 | H-C GopE | 0.003 | 0.021 | 0.888 | 1.000 | 0.888 | No | No |
| 14 | L-C GopE | 0.018 | 0.006 | 0.003 | 0.036 | 0.003 | Yes | Yes |
| 14 | H-P GopE | 0.025 | 0.010 | 0.014 | 0.168 | 0.014 | No | Yes |
| 14 | L-P GopE | 0.015 | 0.008 | 0.065 | 1.000 | 0.065 | No | No |
